# Correction: Getting evidence into clinical practice: protocol for evaluation of the implementation of a home-based cardiac rehabilitation programme for patients with heart failure

**DOI:** 10.1136/bmjopen-2019-036137corr1

**Published:** 2021-03-12

Daw P, van Beurden SB, Greaves C, *et al*. Getting evidence into clinical practice: protocol for evaluation of the implementation of a home-based cardiac rehabilitation programme for patients with heart failure. *BMJ Open* 2020;10:e036137. doi: 10.1136/bmjopen-2019-036137.

This article was previously published with an error.

The paper was linked to an incorrect trial registration number, which has now been removed.

