# Supplementary material for: Getting evidence into clinical practice: protocol for evaluation of the implementation of a home-based cardiac rehabilitation programme for patients with heart failure
Source: BMJ Open. 2020 Jun 21;10(6):e036137. doi: 10.1136/bmjopen-2019-036137 (PMC7307528; doi:10.1136/bmjopen-2019-036137)
Supplement: Supplementary data [file bmjopen-2019-036137supp001.pdf]

What are the barriers and enablers to effective implementation of REACH-HF?

Qualitative interview guide (initial draft*)

** The topic guide content may vary depending on feedback from stakeholders and the first few interviews*

Beacon site: I / II / III / VI *(circle as appropriate)*

Date of interview: ____

- **Welcome and housekeeping**
  Thank you for agreeing to take part in the study. The interview will last between 30 and 40 minutes. I will ask you a series of questions and I am really interested in your honest opinion on the subject matter. If you wish to stop at any point to take a break, let me know.

- **Informed consent**
  Thank you for reading PIS and completing the consent form. Is it ok if we start recording?

- **Interview questions**

NPT	Questions	Comments
1.1	Can you describe REACH-HF intervention and how it differs from your usual way of working?	
1.3	How does the intervention affect the nature of your work?	
4.3	Do you consider it to be worthwhile?	
1.4	In your opinion what is the value of REACH-HF intervention? To you? To your patients?	
1.2	What is your colleagues understanding of the purpose of REACH-HF intervention?	
4.2	Do they consider it to be worthwhile?	
3.2	How has implementing REACH-HF affected working relationships within the team?	
2.1	Who are the individuals (you can include yourself) that drive REACH-HF forward and get others involved? What are their roles? What are they doing to support the project?	
3.1	How easy or difficult has it been to integrate REACH-HF into your existing work?	
2.2	How did the team need to change in order to introduce REACH-HF?	
2.3	How do you feel about being involved in the REACH-HF project?	
3.3	How do the skills of the staff delivering REACH-HF match the needs of the programme?	

REACH-HF beacon sites, Qualitative interview guide, 20.05.2019, version 1, IRAS 261723

What are the barriers and enablers to effective implementation of REACH-HF?

3.4	Was REACH-HF training sufficient to allow for successful implementation? If not, what other topics or skills could have been included?<br><br>Are there enough resources available to support the REACH-HF programme?<br><br>Are there any other **barriers** to delivering REACH-HF on your patch?	
4.1	Are you in any way evaluating effectiveness, usefulness or impact of REACH-HF on the service?	
4.4	Can REACH-HF intervention be modified and improved to suit your way of working? If yes, in what way?	
2.4	What is the future of REACH-HF in your service? What factors can **enable** integration of REACH-HF into a cardiac rehabilitation service?	

- ***A few: service-level questions:*** *What is the catchment area for your service? What population do you serve?*

- **Ending & debrief**
  Thank you for taking the time to answer my questions. Is there anything else you would like to add? Or ask me about? I am going to switch off the audio recorder now. If any of what we spoke about affected you in any way we can have a debrief session now.
