# Supplementary material for: Getting evidence into clinical practice: protocol for evaluation of the implementation of a home-based cardiac rehabilitation programme for patients with heart failure
Source: BMJ Open. 2020 Jun 21;10(6):e036137. doi: 10.1136/bmjopen-2019-036137 (PMC7307528; doi:10.1136/bmjopen-2019-036137)
Supplement: Supplementary data [file bmjopen-2019-036137supp002.pdf]

REACH-HF FIDELITY MEASURE

The rating scale

The seven point scale extends from (0) where the facilitator did not deliver the intervention element appropriately - either they didn't do it well or didn't do it sufficiently (low fidelity) to (6) where there is the element is delivered appropriately (high fidelity). Thus the scale assesses a composite of both adherence to the intended intervention techniques and the skill of the facilitator in delivering the techniques. To aid with the rating of items, an outline of the key features of each item is provided at the top of each section. A generic description of the rating criteria is given in Figure 1.

Adjusting for the presence of patient difficulties

Adjustments may be needed when patient difficulties are evident (e.g. excessive avoidance or resistance). In such circumstances, the rater needs to assess the facilitator's therapeutic skills in the application of the methods. Even though the facilitator may not facilitate change, credit should be given for attempting to use the intended techniques and demonstrating appropriate /skilful interaction (i.e. they should do what they can, within reason, to deliver the intended intervention components).

Figure 1: The scoring system

Competence level*   **Scoring**   **Examples**

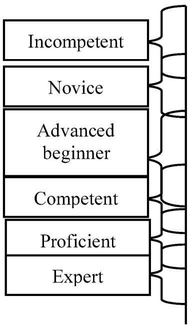

Scoring	Examples
0	Absence of feature and /or highly inappropriate performance
1	Minimal use of feature and /or inappropriate performance,
2	Evidence of competence, but numerous problems
3	Competent, but some problems or inconsistencies
4	Good features, but minor problems or inconsistencies
5	Very good features, minimal problems or inconsistencies
6	Excellent performance

* The scale incorporates the Dreyfus system (Dreyfus, 1989) for denoting competence. Please note that the 'top marks' (i.e. near the 'expert' end of the continuum) are reserved for those facilitators demonstrating highly effective skills, particularly in the face of difficulties (i.e. patients with high resistance to change; high levels of emotional expression; and complex situational barriers). Please note that there are 5 competence levels but six potential scores.

When rating the item, you should first identify whether some of the 'Key Features' are present. If the facilitator includes most of the key features and uses them appropriately (i.e. misses few relevant opportunities to use them and delivers them well), the facilitator should be rated highly. It is important to remember that the scoring profile for this scale should approximate to a normal distribution, with relatively few people

Daw P, *et al. BMJ Open* 2021; 10:e036137. doi: 10.1136/bmjopen-2019-036137

scoring at the extremes. **For the purposes of the REACH study, a score of 3 or more will be taken to represent "acceptable delivery or basic competence" in using the intended techniques"**

Dreyfus, H. L. (1989). The Dreyfus model of skill acquisition. In J. Burke (ed.) Competency based education and training. London: Falmer Press.

ITEM 1: ACTIVE PATIENT INVOLVEMENT

Key features: The facilitator should encourage the participant to be actively involved in the consultation. The idea is to maximise the participant's autonomy as the main agent of change, developing intrinsic rather than extrinsic motivation, and encouraging her /him to be the person coming up with ideas for improving the situation. However, the participant should not be allowed to ramble in an unstructured way and the consultation should be guided. A collaborative /shared decision-making style is appropriate and the facilitator may share his /her own expertise and ideas (as below). Overall, the participant should be increasingly empowered to take control of her /his self care behaviour. Interactions should be encouraging, respectful and non-judgemental (the opposite of a didactic, telling or persuading style of interaction). The participant should ideally talk for at least half of the time (particularly in later sessions). The interaction should also be *individually tailored* to the patient's specific information needs, beliefs, motivations and barriers. The facilitator should engender a clear sense of warmth, genuineness and empathy (within professional boundaries).

Intervention techniques: OARS (Open questions, Affirmation, Reflective listening, Summaries). Reflective listening may include simple reflections of content but may also be more sophisticated (e.g. amplified reflection; reflection with a twist) and used to direct the conversation or highlight key strengths or barriers. Summaries to reinforce patient choices and acknowledge patient effort are particularly desirable. Individual tailoring of techniques and responses to the individual patient's existing knowledge, skills, current activity levels, needs and preferences are also desirable. The Ask-Tell-Discuss technique should be used to exchange information (e.g. to address misconceptions, or offer helpful new information). The above empathy-building techniques and individual tailoring should be used throughout the consultations - from the initial consultation through action-planning through to review /maintenance sessions.

Mark with an 'X' on the vertical line, using whole and half numbers, the level to which you think the facilitator has delivered this intervention process

0 Absence of active patient involvement techniques. An overly 'directing', practitioner-led or 'lecturing' style of interaction, which may increase or sustain client's resistance.

1 Minimal patient involvement or use of active patient involvement techniques. The practitioner dominates the discussion.

2 Some use of patient involvement techniques, but not frequent enough. The practitioner sometimes dominates the discussion.

3 Appropriate and frequent use of patient involvement techniques. Teamwork evident, but some difficulties in content or method of delivery.

4 Appropriate and frequent use of patient involvement techniques. Minor problems evident (e.g. some reflection opportunities missed).

Daw P, et al. BMJ Open 2021; 10:e036137. doi: 10.1136/bmjopen-2019-036137

5 Highly appropriate and regular use of patient involvement techniques, facilitating shared understanding and decision making. Minimal problems.

6 Excellent / expert use of patient involvement techniques throughout all the consultation. A clear sense of collaborative alliance is developed.

Daw P, *et al. BMJ Open* 2021; 10:e036137. doi: 10.1136/bmjopen-2019-036137

ITEM 2: ASSESSING THE PATIENT'S CURRENT SITUATION AND NEEDS.

Key features: The facilitator should work with the participant to assess the patient's current situation. They should seek to identify ALL of the following over the first 1-2 sessions: Identify and discuss the most important issue currently for the patient, how well are they managing their fluids, how appropriately are they using medications, is there any obvious immediate clinical need, how much stress or anxiety do they have, how much physical activity are they doing, and what other concerns or questions they may have.

Intervention techniques: Facilitators will use patient-centred communication techniques (as above) which may include the Ask-Tell-Discuss and 'tell me three things' technique to explore the patient's current situation.

Mark with an 'X' on the vertical line, using whole and half numbers, the level to which you think the facilitator has delivered this intervention process

0 Absence (or very poor delivery) of discussions to assess the patient's current situation.

1 Minimal (or poorly delivered) discussions to assess the patient's current situation.

2 Some discussions to assess the patient's current situation, but may not be in sufficient depth or detail, or quality of delivery may be variable.

3 Several examples of discussion to assess the patient's current situation. However some difficulties evident (e.g. missed opportunities, not covering all the key topics, or talking at odds with the patient).

4 Several examples of discussion to assess the patient's current situation. Minor problems evident.

5 Highly appropriate and sufficient discussion to assess the patient's current situation. Minimal problems.

6 Excellent / expert use of discussion to assess the patient's current situation. No real problems.

Daw P, *et al. BMJ Open* 2021; 10:e036137. doi: 10.1136/bmjopen-2019-036137

ITEM 3: FORMULATING AN APPROPRIATE (INDIVIDUALISED) TREATMENT PLAN

Key features: The facilitator should work with the participant to formulate an appropriate treatment plan based on the patient's current situation. This should aim to address (as a minimum) ALL of the following over the twelve weeks of the programme: What is the most important issue currently for the patient, are they managing their fluids well, are they using medications appropriately, any clinical needs identified, how much stress or anxiety do they have, how much physical activity are they doing, and any other concerns or questions they may have. The treatment plan will be staged over time, aiming to work on a few topics initially and introducing other elements as the programme continues. It is best practice to summarise the treatment plan at the end of the session "what we have said today is …".

Intervention techniques: Facilitators will use patient-centred communication techniques (as above) to discuss and agree what issues to address first and what order to do things in. An element of guiding to ensure the inclusion of clinical priorities (e.g. medication issues, physical activity, psychological well-being) as well as patient priorities may be appropriate. The facilitator will advise the patient (and caregiver if appropriate) to read relevant sections of the manual ahead of their next meeting.

Mark with an 'X' on the vertical line, using whole and half numbers, the level to which you think the facilitator has delivered this intervention process

0 Absence (or very poor delivery) of discussion to formulate an appropriate treatment plan based on the patient's current situation.

1 Minimal (or poorly delivered) discussion to formulate an appropriate treatment plan based on the patient's current situation.

2 Some discussion to formulate an appropriate treatment plan based on the patient's current situation, but may not be in sufficient depth or detail, or quality of delivery may be variable (e.g. not covering all the key topics, or talking at odds with the patient).

3 Several examples of discussion to formulate an appropriate treatment plan based on the patient's current situation. However some difficulties may still be evident (e.g. missed opportunities, plan not summarised at the end of the visit).

4 Several examples of discussion to formulate an appropriate treatment plan based on the patient's current situation. Minor problems evident.

5 Highly appropriate and sufficient discussion to formulate an appropriate treatment plan based on the patient's current situation. Minimal problems.

6 Excellent / expert use of discussion to formulate an appropriate treatment plan based on the patient's current situation. No real problems.

Daw P, *et al. BMJ Open* 2021; 10:e036137. doi: 10.1136/bmjopen-2019-036137

ITEM 4: BUILD THE PATIENT'S UNDERSTANDING OF HEART FAILURE /MAKING A LINK BETWEEN SELF-CARE ACTIVITIES AND THEIR HEART FAILURE SYMPTOMS

Key features: Participants' ability to make sense of how HF works and how self-care behaviours might influence the course of the illness will be crucial for the success of the intervention as belief in the benefit of the suggested self-care activities will increase motivation to engage in them. The facilitator should elicit the patient's current understanding of heart failure and seek to build their 'illness model' in terms of understanding the Identity, Causes, Consequences, Cure /control options and Timeline[1] associated with the condition. This process may take several weeks and should be reinforced as the programme progresses.

Intervention techniques: Facilitators will provide the REACH-HF Manual, provide a brief overview of how the manual works and, after assessing the patient's individual needs and concerns (as above), they will identify some key sections for the patient to read before the next contact, specifically including the Understanding HF section. Facilitators will use patient-centred communication techniques (as above) to elicit and build understanding. This should include the use of the Ask-Tell-Discuss technique and reflective listening to reinforce elements of the patient's understanding that are factually correct or which predispose towards positive self-care behaviours. They should seek to reframe negative attitudes and exchange information (Ask-Tell-Discuss) to address any misconceptions or to fill any important gaps in understanding. The facilitator will advise the patient (and caregiver if appropriate) to read relevant sections of the manual (including the Understanding HF chapter) to build and reinforce understanding /to address misconceptions. The way HF works should be explicitly discussed and referred back to /reinforced at subsequent sessions when this reinforces perceived benefits of the proposed self-care behaviours.

Mark with an 'X' on the vertical line, using whole and half numbers, the level to which you think the facilitator has delivered this intervention process.

0 Absence (or very poor delivery) of any exploration or discussion of how HF works. Understanding of HF is assumed or not mentioned or discussed.

1 Minimal (or poor delivery of) exploration or discussion of how HF works.

2 Some exploration or discussion of the how HF works, but may not be in sufficient depth or detail, or quality of delivery may be variable (e.g. telling rather than Ask-Tell-Discuss) or understanding is not checked.

3 Appropriate exploration and discussion of how HF works. However, some difficulties may still be evident (e.g. moving on before understanding is fully established).

4 Appropriate exploration or discussion of how HF works, linking changes in symptoms or mood with changes in self-care behaviour. Minor problems evident (e.g. some inconsistencies).

5 Highly appropriate and sufficient exploration or discussion of how HF works, facilitating a clear understanding of the process and linking changes in symptoms and mood with changes in self-care behaviour. Minimal problems.

6 Excellent / expert exploration and discussion facilitating a clear understanding of how HF works and the reasons for change. No real problems.

1. Leventhal H, Nerenz DR, Steele DJ: Illness representations and coping with health threats. In: *Handbook of Psychology and Health*. Volume IV. Edited by Baum AE, et al. Hillsdale NJ: Lawrence Erlbaum; 1984: 219-67.

Daw P, *et al*. *BMJ Open* 2021; 10:e036137. doi: 10.1136/bmjopen-2019-036137

ITEM 5a: SUPPORTING SELF-MONITORING AND PROGRESS-TRACKING

Key features: The facilitator should agree a verbal plan of action for the following week(s) with the patient. and discuss the use of the progress-tracking tools in the HF Manual to keep track of progress and as a way of recording any problems in completing the activities and any benefits that might be associated with the planned activities.

Intervention techniques: The facilitator should encourage the participant to monitor /keep track of their activities using the progress-tracking tools in the HF Manual.

Mark with an 'X' on the vertical line, using whole and half numbers, the level to which you think the facilitator has delivered this intervention process

0 Absence (or very poor delivery) of encouragement of self-monitoring.

1 Minimal (or poorly delivered) encouragement of self-monitoring. Activities planned are not sustainable, or poorly specified.

2 Some encouragement of self-monitoring but lacking detail /patient involvement in the activity may be limited, or quality of delivery may be variable (e.g. telling rather than discussing).

3 Appropriate encouragement of self-monitoring. However, some difficulties evident (e.g. not explaining the rationale for using the tool as a basis for monitoring progress, sometimes providing rather than eliciting ideas).

4 Appropriate encouragement of self-monitoring. Minor problems evident (e.g. the plan is a bit less specific than it could be).

5 Highly appropriate encouragement of self-monitoring. The participant has a clear understanding of the plan for the week ahead and how to monitor progress. Minimal problems

6 Excellent / expert encouragement of self-monitoring. The participant has a clear and realistic understanding of how to monitor progress. No real problems.

Daw P, *et al. BMJ Open* 2021; 10:e036137. doi: 10.1136/bmjopen-2019-036137

ITEM 5b: REVIEWING PROGRESS AND PROBLEM-SOLVING

Key features: The facilitator should work with the participant to review progress with all planned changes and with achieving the targets set out in the action plan. The facilitator should celebrate and reinforce and reflect on any successes. The participant and facilitator should discuss any setbacks and the patient's plans should be revised.

Intervention techniques: The facilitator should reinforce any self-monitoring activity and any successes in behaviour change (by giving praise/ using Affirmation techniques). Reframing should be used to normalise setbacks and see them as an opportunity to learn from experience (trial and error) rather than as failures. Problem-solving should use OARS (Open questions, Affirmation, Reflective listening, Summaries) and information exchange (Ask-Tell-Discuss) techniques to identify barriers and explore ways to overcome them. Problem-solving may specifically focus on issues of connectedness (social influences, involvement of others in supporting activities) and sustainability, or on breaking the problem down into more manageable chunks. Goals /action plans should be reviewed and revised if necessary.

Mark with an 'X' on the vertical line, using whole and half numbers, the level to which you think the facilitator has delivered this intervention process

0 Absence (or very poor delivery) of any progress review. No reinforcement of success and discussion of setbacks or barriers in relation to the previous weeks planned activities /problem-solving, or reviewing action plans.

1 Minimal (or poor delivery) of progress review. Minimal reinforcement of success and discussion of setbacks or barriers in relation to the previous weeks planned activities /problem-solving, or reviewing action plans.

2 Some progress review. Some reinforcement of success and discussion of setbacks or barriers in relation to the previous weeks planned activities /problem-solving and reviewing action plans, but lacking sufficient depth or detail or may be poorly delivered (e.g. providing solutions rather than using Ask-Tell-Discuss).

3 Appropriate progress review. Appropriate reinforcement of success and discussion of setbacks or barriers in relation to the previous weeks planned activities /problem-solving, and reviewing action plans. However, some difficulties evident (e.g. not reframing setbacks, not attempting to identify problems, or possible solutions).

4 Appropriate progress review. Appropriate reinforcement of success and discussion of setbacks or barriers in relation to the previous weeks planned activities /problem-solving, and reviewing action plans. Minor problems evident.

5 Highly appropriate and sufficient progress review. Appropriate reinforcement of success and discussion of setbacks or barriers in relation to the previous weeks planned activities /problem-solving, or reviewing action plans. Minimal problems.

Daw P, *et al. BMJ Open* 2021; 10:e036137. doi: 10.1136/bmjopen-2019-036137

6 Excellent / expert progress review. Excellent reinforcement of success and discussion of setbacks or barriers in relation to the previous weeks planned activities /problem-solving, and reviewing action plans. No real problems.

Daw P, *et al. BMJ Open* 2021; 10:e036137. doi: 10.1136/bmjopen-2019-036137

ITEM 6: MAKE A SPECIFIC ACTION PLAN FOR PHYSICAL ACTIVITY, BASED ON THE ACTIVITIES SELECTED BY THE PATIENT

Key features: Using the template in the HF manual, the facilitator should work with the participant to agree a written or verbal plan of action for engaging in one of the physical activity /exercise options over the following week(s). This should include discussion to ensure an appropriate intensity (moderate) of any activity included in the action plan.

Intervention techniques: Making a written action plan, using the planning tool in the manual, or a verbal action plan for physical activity. The facilitator should ensure that goal-setting is realistic. The facilitator may also employ some problem-solving techniques at this stage to pre-empt and address potential problems. It is best practice to summarise the plan at the end of the session “what we have said today is …”.

Mark with an 'X' on the vertical line, using whole and half numbers, the level to which you think the facilitator has delivered this intervention process

0 Absence (or very poor delivery) of activity /exercise planning for the following week(s).

1 Minimal use (or poor delivery) of activity /exercise planning for the following week(s). Activities planned are not sustainable, or representative of the routine, pleasurable and necessary activities previously identified.

2 Some use of action-planning techniques using the HF Manual planning tool (or verbal equivalent) but lacking detail /patient involvement in the activity may be limited. Quality of delivery may be variable (e.g. providing the plan rather than discussing, not checking the patient is happy with the plan).

3 Appropriate use of action planning techniques . However, some difficulties evident (e.g. not summarising the plan at the end, sometimes providing rather than eliciting ideas).

4 Appropriate use of action planning techniques. Minor problems evident (e.g. the plan is a bit less specific than it could be).

5 Highly appropriate and sufficient use of action-planning techniques. The participant has a clear understanding of and ownership of the plan for the week(s) ahead. Minimal problems.

6 Excellent / expert use of action-planning techniques. The participant has a clear understanding of the rationale behind planning for the week(s) ahead, and has a clear and realistic action plan for the week(s) ahead. No real problems.

Daw P, *et al. BMJ Open* 2021; 10:e036137. doi: 10.1136/bmjopen-2019-036137

ITEM 7: ADDRESSING EMOTIONAL CONSEQUENCES OF HEART FAILURE

Key features: The facilitator should help the patient to recognise and address any significant stress, anxiety, anger, depression or other negative feelings that are related to having heart failure. S/he should seek to normalise such feelings and help the patient to access and work through relevant sections of the manual. If these problems are severe or prolonged the facilitator should facilitate a referral to relevant care services.

Intervention techniques: Patient centred counselling techniques (OARS) for assessment and exchanging information to build patient's understanding of the situation. Facilitation of the cognitive behavioural therapy techniques and stress management techniques contained within the manual.

Mark with an 'X' on the vertical line, using whole and half numbers, the level to which you think the facilitator has delivered this intervention process

0 Absence (or very poor delivery) of any attempts to address emotional consequences.

1 Minimal (or poorly delivered) attempts to address emotional consequences,

2 Some attempts to address emotional consequences, but lacking sufficient depth or detail. Quality of delivery may be variable (e.g. talking at odds with the patient).

3 Appropriate attempts to address emotional consequences. However, some difficulties evident (e.g. sometimes being prescriptive rather than patient-centred, not identifying all relevant sections of the manual).

4 Appropriate attempts to address emotional consequences. Minor problems evident.

5 Highly appropriate and sufficient addressing of emotional consequences. Minimal problems.

6 Excellent / expert addressing of emotional consequences. No real problems.

Daw P, *et al. BMJ Open* 2021; 10:e036137. doi: 10.1136/bmjopen-2019-036137

ITEM 8: ADDRESSING MEDICATION ISSUES

Key features: The facilitator should help the patient to recognise and address any significant problems or concerns relating to the patient's heart failure medications. S/he should help the patient to work through relevant sections of the manual. This might include problems in organising /taking the medications, knowing what to do if they get a cold or forget a dose, identifying possible side effects and seeking help to minimise them, avoiding over-the-counter medications. For some patients, it may include discussing self-titration of diuretics (water tablets) in response to symptoms /swelling (using the Traffic Light plan as a guide).

Intervention techniques: Patient centred counselling techniques (OARS) for assessment and to exchange information to build patient's understanding of the situation. Facilitation of medication planning /monitoring tools (in the Progress Tracker) and tips provided in the manual.

Mark with an 'X' on the vertical line, using whole and half numbers, the level to which you think the facilitator has delivered this intervention process

0 Absence (or very poor delivery) of any attempts to address medication issues.

1 Minimal (or poor delivery) attempts to address medication issues.

2 Some attempts to address medication issues, but lacking sufficient depth or detail, or quality of delivery may be variable (e.g. not picking up /addressing concerns about possible side effects)

3 Appropriate attempts to address medication issues. However, some difficulties evident (e.g. sometimes being prescriptive rather than patient-centred, not identifying all relevant sections of the manual).

4 Appropriate attempts to address medication issues. Minor problems evident.

5 Highly appropriate and sufficient addressing of medication issues. Minimal problems.

6 Excellent / expert addressing of medication issues. No real problems.

Daw P, *et al. BMJ Open* 2021; 10:e036137. doi: 10.1136/bmjopen-2019-036137

ITEM 9: CAREGIVER INVOLVEMENT (as applicable)

Key features: The facilitator should engage the caregiver as much as possible as a co-facilitator of the intervention. S/he should tailor the intervention to work with the caregiver's abilities and availability to provide support to the cared for person with self-management of their heart failure. Facilitators will provide the Caregiver Resource, a brief overview of what it contains, and identify some key sections for the caregiver to read.

Intervention techniques: Person centred counselling techniques (OARS) for assessment and to exchange information to build the caregiver's understanding of the situation and their ability to support the person with heart failure with their self-management. The facilitator should facilitate a conversation between the patient and the caregiver to agree their roles and responsibilities and how these might change if the patient's condition declines. Attention should be given to the caregiver's needs and concerns about being a caregiver /providing care as well as those of the patient.

Mark with an 'X' on the vertical line, using whole and half numbers, the level to which you think the facilitator has delivered this intervention process

0 Absence (or very poor delivery) of any attempts to involve the caregiver or to address his /her needs.

1 Minimal (or poor delivery) attempts to involve the caregiver or to address his /her needs.

2 Some attempts to involve the caregiver or to address his /her needs, but lacking sufficient depth or detail, or quality of delivery may be variable (e.g. being mostly prescriptive rather than person-centred).

3 Appropriate attempts to involve the caregiver or to address his /her needs. However, some difficulties evident (e.g. leaving roles and responsibilities between patient and caregiver unclear in some respects).

4 Appropriate attempts to involve the caregiver or to address his /her needs. Minor problems evident.

5 Highly appropriate and sufficient involvement of the caregiver and addressing his /her needs. Minimal problems.

6 Excellent / expert involvement of the caregiver and addressing his /her needs. No real problems.

Daw P, et al. BMJ Open 2021; 10:e036137. doi: 10.1136/bmjopen-2019-036137

ITEM 10: ADDRESSING EMOTIONAL CONSEQUENCES OF BEING A CAREGIVER (as applicable)

Key features: The facilitator should help the caregiver to recognise and address any significant stress, anxiety, anger, depression or other negative feelings that are related to becoming a caregiver and supporting someone with heart failure. S/he should seek to normalise such feelings and help the caregiver to access and work through relevant sections of the Caregiver Resource. This includes facilitating a referral for a carer's assessment if the caregiver wishes, plus referral to other relevant care services as appropriate.

Intervention techniques: Person centred counselling techniques (OARS) for assessment and to exchange information to build the caregiver's understanding of the situation. Facilitation of the cognitive behavioural therapy techniques and stress management techniques contained within the manual.

Mark with an 'X' on the vertical line, using whole and half numbers, the level to which you think the facilitator has delivered this intervention process

0 Absence (or very poor delivery) of any attempts to address emotional consequences.

1 Minimal (or poorly delivered) attempts to address emotional consequences.

2 Some attempts to address emotional consequences, but lacking sufficient depth or detail, or quality of delivery may be variable (e.g. talking at odds with the patient).

3 Appropriate attempts to address emotional consequences. However, some difficulties evident (e.g. sometimes being prescriptive rather than patient-centred, not identifying all relevant sections of the manual, not facilitating onward referrals).

4 Appropriate attempts to address emotional consequences. Minor problems evident.

5 Highly appropriate and sufficient addressing of emotional consequences. Minimal problems.

6 Excellent / expert addressing of emotional consequences. No real problems.

Daw P, *et al. BMJ Open* 2021; 10:e036137. doi: 10.1136/bmjopen-2019-036137

Daw P, *et al. BMJ Open* 2021; 10:e036137. doi: 10.1136/bmjopen-2019-036137

ITEM 11: CAREGIVER HEALTH AND WELL-BEING (as applicable)

Key features: The facilitator should help the caregiver to prioritise and look after their own health and well-being.

Intervention techniques: Person centred counselling techniques (OARS) for assessment and to exchange information to build the caregiver's understanding of the situation – helping them recognise and manage their own health needs including mental health, physical health, and social needs. This may be a separate conversation with the caregiver alone.

Mark with an 'X' on the vertical line, using whole and half numbers, the level to which you think the facilitator has delivered this intervention process

0 Absence (or very poor delivery) of any attempts to involve the caregiver or to address his /her health needs.

1 Minimal (or poor delivery of) attempts to involve the caregiver or to address his /her health needs.

2 Some attempts to involve the caregiver or to address his /her needs, but lacking sufficient depth or detail, or quality of delivery may be variable (e.g. not picking up on /addressing some of the caregiver's concerns).

3 Appropriate attempts to involve the caregiver or to address his /her needs. However, some difficulties evident (e.g. sometimes being prescriptive rather than patient-centred, failing to identify the appropriate sections of the Caregiver's Resource).

4 Appropriate attempts to involve the caregiver or to address his /her needs. Minor problems evident.

5 Highly appropriate and sufficient involvement of the caregiver and addressing his /her needs. Minimal problems.

6 Excellent / expert involvement of the caregiver and addressing his /her needs. No real problems.

Daw P, *et al. BMJ Open* 2021; 10:e036137. doi: 10.1136/bmjopen-2019-036137

ITEM 12: BRINGING THE PROGRAMME TO A CLOSE

Key features: Progress should be consolidated and reinforced. Plans for long-term sustainability of activities and strategies learned for managing heart failure should be discussed.

Intervention techniques: The facilitator will review progress since the start of the intervention and reinforce what has been learnt. Useful strategies that were helpful should be identified. Plans to stay well /prevent relapse should be discussed as well as 'cues for action' and plans to revisit the manual in the future. The facilitator will discuss plans to sustain any new activities, identifying any potential problems and coping strategies to overcome these. The possibility of good and bad days should be discussed and normalised.

Mark with an 'X' on the vertical line, using whole and half numbers, the level to which you think the facilitator has delivered this intervention process

0 Absence (or very poor delivery) of discussion to bring the intervention to a close. Not considering progress and long term planning using the above strategies.

1 Minimal (or poorly delivered) discussion to bring the intervention to a close. Minimal consideration of progress and long term planning using the above strategies.

2 Some discussion to bring the intervention to a close. Some consideration of progress and long term planning using the above strategies, but not in sufficient depth or detail, or quality of delivery may be variable (e.g. telling /providing solutions rather than discussing or eliciting solutions from the patient (and caregiver if relevant)).

3 Appropriate discussions to bring the intervention to a close. Appropriate consideration of progress and long term planning using the above strategies. However some difficulties evident (e.g. missed opportunities to reinforce what has been learnt, facilitator sometimes dominating the conversation /telling rather than facilitating development of the patient's own ideas).

4 Several examples of appropriate discussion to bring the intervention to a close and examples of consideration of progress and long term planning the above strategies. Minor problems evident.

Daw P, *et al. BMJ Open* 2021; 10:e036137. doi: 10.1136/bmjopen-2019-036137

5 Highly appropriate and sufficient discussion to bring the intervention to a close and to consider progress and long term planning using the above strategies. Minimal problems.

6 Excellent / expert discussions to bring the intervention to a close and to consider progress and long term planning using the above strategies. No real problems.

Daw P, *et al. BMJ Open* 2021; 10:e036137. doi: 10.1136/bmjopen-2019-036137

CONTENT CHECKLIST - PATIENT

**How much did the facilitator cover the following topics in this session with regard to the patient…**	**Not at all <- Partially -> Thoroughly**				
1. … Understanding heart failure	1	2	3	4	5
2. ... Management of stress or anxiety	1	2	3	4	5
3. ... Physical activity	1	2	3	4	5
4. ... Low mood /depression	1	2	3	4	5
5. … Taking medications	1	2	3	4	5
6. ... Deciding priorities/ setting goals					
7. … Tracking and reviewing progress	1	2	3	4	5
8. ... Using the HF Manual	1	2	3	4	5
9. ...Support from others	1	2	3	4	5
10. … Other (please state)	1	2	3	4	5

Daw P, *et al. BMJ Open* 2021; 10:e036137. doi: 10.1136/bmjopen-2019-036137

CONTENT CHECKLIST - CAREGIVER

**How much did the facilitator cover the following topics in this session with regard to the caregiver …**	**Not at all**		**<- Partially ->**		**Thoroughly**
1. ... Assessing the caregiver's needs<br>e.g. understanding of HF, how to facilitate self care	1	2	3	4	5
2. ... Managing the caregiver's own health and well-being	1	2	3	4	5
3. ... Facilitating discussion of /decisions about care-giving roles and responsibilities	1	2	3	4	5
4. ... Promoting physical activity for the patient	1	2	3	4	5
5. ...Encouraging self-monitoring and management for the patient	1	2	3	4	5
6. ... Helping patients who feel stressed or depressed	1	2	3	4	5
7. … Understanding and managing the patient's medications	1	2	3	4	5
8. … Other (please state)<br>e.g. financial management, getting help from friends, uncertainty	1	2	3	4	5

Leventhal H, Nerenz DR, Steele DJ: Illness representations and coping with health threats. In: *Handbook of Psychology and Health*. Volume IV. Edited by Baum AE, et al. Hillsdale NJ: Lawrence Erlbaum; 1984: 219-67.

Daw P, *et al. BMJ Open* 2021; 10:e036137. doi: 10.1136/bmjopen-2019-036137
