# Supplementary material for: Getting evidence into clinical practice: protocol for evaluation of the implementation of a home-based cardiac rehabilitation programme for patients with heart failure
Source: BMJ Open. 2020 Jun 21;10(6):e036137. doi: 10.1136/bmjopen-2019-036137 (PMC7307528; doi:10.1136/bmjopen-2019-036137)
Supplement: Supplementary data [file bmjopen-2019-036137supp003.pdf]

Dear REACH-HF facilitator,

At the end of each REACH-HF session that you have audio recorded, we would like you to take a few moments to reflect on how the session went. Each line on the checklist represents a key feature of the programme. You can rate the session from 0 to 6, where 0 means that you did not use the particular feature of the programme and 6 means that you used such feature extensively and proficiently.
There is no right or wrong way to answer these questions and your or your team's performance will not be judged in any way. We appreciate that some features will be more relevant at different points of the treatment and we do not expect you to include all features in every session. Your honesty will be greatly appreciated.

Session date: ____________________ Participant study number: ____________________ Session number: __________________

**REACH-HF programme feature**	Absence	Minimal	Some	Sufficient	Good	Very good	Excellent
1. Active patient involvement	0	1	2	3	4	5	6
2. Assessing the patient's current situation and needs	0	1	2	3	4	5	6
3. Formulating an appropriate (individualised) treatment plan	0	1	2	3	4	5	6
4. Building the patient's understanding of heart failure /making a link between self-care activities and their heart failure symptoms	0	1	2	3	4	5	6
5a. Supporting self-monitoring and progress-tracking	0	1	2	3	4	5	6
5b. Reviewing progress and problem-solving	0	1	2	3	4	5	6
6. Making a specific action plan for physical activity, based on the activities selected by the patient	0	1	2	3	4	5	6
7. Addressing emotional consequences of heart failure	0	1	2	3	4	5	6
8. Addressing medication issues	0	1	2	3	4	5	6
9. Caregiver involvement (as applicable)	0	1	2	3	4	5	6
10. Addressing emotional consequences of being a caregiver (as applicable)	0	1	2	3	4	5	6
11. Caregiver health and well-being (as applicable)	0	1	2	3	4	5	6
12. Bringing the programme to a close	0	1	2	3	4	5	6

REACH-HF beacon sites, Self-rated fidelity checklist, 16.05.2019, version 1, IRAS 261723
