# Supplementary material for: Getting evidence into clinical practice: protocol for evaluation of the implementation of a home-based cardiac rehabilitation programme for patients with heart failure
Source: BMJ Open. 2020 Jun 21;10(6):e036137. doi: 10.1136/bmjopen-2019-036137 (PMC7307528; doi:10.1136/bmjopen-2019-036137)
Supplement: Author's manuscript [file bmjopen-2019-036137.draft_revisions.pdf]

BMJ Open is committed to open peer review. As part of this commitment we make the peer review history of every article we publish publicly available.

When an article is published we post the peer reviewers' comments and the authors' responses online. We also post the versions of the paper that were used during peer review. These are the versions that the peer review comments apply to.

The versions of the paper that follow are the versions that were submitted during the peer review process. They are not the versions of record or the final published versions. They should not be cited or distributed as the published version of this manuscript.

BMJ Open is an open access journal and the full, final, typeset and author-corrected version of record of the manuscript is available on our site with no access controls, subscription charges or pay-per-view fees (http://bmjopen.bmj.com).

If you have any questions on BMJ Open's open peer review process please email info.bmjopen@bmj.com

BMJ Open

Getting evidence into clinical practice: Protocol for evaluation of the implementation of a home-based cardiac rehabilitation programme for patients with heart failure.

Journal:	*BMJ Open*
Manuscript ID	bmjopen-2019-036137
Article Type:	Protocol
Date Submitted by the Author:	02-Dec-2019
Complete List of Authors:	Daw, Paulina; University of Birmingham, School of Sport, Exercise & Rehabilitation Sciences<br>van Beurden, Samantha; University of Exeter, Sport and Health Sciences; University of Bristol, School of Experimental Psychology<br>Greaves, Colin; University of Birmingham, School of Sport, Exercise & Rehabilitation Sciences<br>Veldhuijzen van Zanten, Jet; University of Birmingham, School of Sport, Exercise and Rehabilitation Sciences<br>Harrison, Alexander; University of York, Health Sciences<br>Dalal, Hasnain; Royal Cornwall Hospitals NHS Trust; University of Exeter, Medical School<br>McDonagh, Sinead; University of Exeter, Medical School, Primary Care Department<br>Doherty, Patrick Joseph; University of York, Health Sciences<br>Taylor, Rod; University of Glasgow, MRC/CSO Social and Public Health Sciences Unit & Robertson Centre for Biostatistics, Institute of Health and Well Being; University of Exeter, Exeter Clinical Trials Unit, College of Medicine and Health
Keywords:	Heart failure < CARDIOLOGY, REHABILITATION MEDICINE, Change management < HEALTH SERVICES ADMINISTRATION & MANAGEMENT

SCHOLARONE™
Manuscripts

I, the Submitting Author has the right to grant and does grant on behalf of all authors of the Work (as defined in the below author licence), an exclusive licence and/or a non-exclusive licence for contributions from authors who are: i) UK Crown employees; ii) where BMJ has agreed a CC-BY licence shall apply, and/or iii) in accordance with the terms applicable for US Federal Government officers or employees acting as part of their official duties; on a worldwide, perpetual, irrevocable, royalty-free basis to BMJ Publishing Group Ltd ("BMJ") its licensees and where the relevant Journal is co-owned by BMJ to the co-owners of the Journal, to publish the Work in this journal and any other BMJ products and to exploit all rights, as set out in our licence.

The Submitting Author accepts and understands that any supply made under these terms is made by BMJ to the Submitting Author unless you are acting as an employee on behalf of your employer or a postgraduate student of an affiliated institution which is paying any applicable article publishing charge ("APC") for Open Access articles. Where the Submitting Author wishes to make the Work available on an Open Access basis (and intends to pay the relevant APC), the terms of reuse of such Open Access shall be governed by a Creative Commons licence – details of these licences and which Creative Commons licence will apply to this Work are set out in our licence referred to above.

Other than as permitted in any relevant BMJ Author's Self Archiving Policies, I confirm this Work has not been accepted for publication elsewhere, is not being considered for publication elsewhere and does not duplicate material already published. I confirm all authors consent to publication of this Work and authorise the granting of this licence.

For peer review only

Getting evidence into clinical practice: Protocol for evaluation of the implementation of a home-based cardiac rehabilitation programme for patients with heart failure.

Paulina Daw (1st author and corresponding author)
School of Sport, Exercise & Rehabilitation Sciences
University of Birmingham
Edgbaston
B15 2TT
Mobile: 0759 555 0720
Email address: pxd891@student.bham.ac.uk
ORCID iD: 0000-0002-0942-3953

Samantha van Beurden (1st author)
Sport and Health Sciences
University of Exeter
Exeter, UK
&
School of Experimental Psychology
University of Bristol
Bristol, UK

PD and SVB contributed equally to this paper.

Co-authors:
Colin J Greaves
School of Sport, Exercise & Rehabilitation Sciences
University of Birmingham
Birmingham, UK

Jet JCS Veldhuijzen van Zanten
School of Sport, Exercise & Rehabilitation Sciences
University of Birmingham
Birmingham, UK

Alexander Harrison
Health Sciences
University of York, UK

Hasnain Dalal
University of Exeter Medical School
Exeter, UK
&
Royal Cornwall Hospitals NHS Trust
Cornwall, UK

Sinead TJ McDonagh
Primary Care Department
University of Exeter Medical School
Exeter, UK

For peer review only

Patrick J Doherty
Health Sciences
University of York
York, UK

Rod Taylor
MRC/CSO Social and Public Health Sciences Unit & Robertson Centre for Biostatistics
Institute of Health and Well Being
University of Glasgow
Glasgow, UK
&
Exeter Clinical Trials Unit
College of Medicine and Health
University of Exeter
Exeter, UK

Word count: 3872

For peer review only

ABSTRACT

Introduction: Cardiac rehabilitation (CR) improves health-related quality of life (HRQoL) and reduces hospital admissions. However, heart failure (HF) patients often fail to attend centre-based CR programmes. Novel ways of delivering healthcare, such as home-based CR programmes, may improve uptake of CR. Rehabilitation EnAblement in CHronic Heart Failure (REACH-HF) is a new, effective and cost-effective home-based CR programme for people with HF.

The aim of this prospective mixed-methods implementation evaluation study is to assess the implementation of the REACH-HF CR programme in the United Kingdom (UK) National Health Service (NHS). The specific objectives are to a) explore NHS staff perceptions of the barriers and facilitators to the implementation of REACH-HF, b) assess the quality of delivery of the programme in real-life clinical settings c) consider the nature of any adaptation(s) made and how they might impact on intervention effectiveness, and d) compare real-world patient outcomes to those seen in a prior clinical trial.

Methods and analysis: REACH-HF will be rolled-out in four NHS CR centres across the UK. Three healthcare professionals from each site will be trained to deliver the 12-week programme. In-depth qualitative interviews and focus groups will be conducted with approximately 24 NHS professionals involved in delivering or commissioning the programme. Consultations for 48 patients (12 per site) will be audio-recorded and scored using an intervention fidelity checklist. Outcomes routinely recorded in the National Audit of Cardiac Rehabilitation will be analysed and compared with outcomes from a recent randomised controlled trial: the Minnesota Living with HF Questionnaire and exercise capacity (Incremental Shuttle Walk Test). Qualitative research findings will be mapped onto the Normalisation Process Theory framework and presented in the form of a narrative synthesis. Results of the study will inform national roll-out of REACH-HF.

ARTICLE SUMMARY

Ethics and dissemination: The study (IRAS 261723) has received ethics approval from the South Central (Hampshire B) Research Ethics Committee (19/SC/0304). The findings will be published in peer-reviewed journals and presented at conferences.

Keywords: cardiac rehabilitation, heart failure, implementation science, Normalisation Process Theory.

Strengths and limitations of this study:

- This will be the first study to investigate the real-world implementation of a home-based cardiac rehabilitation programme in the UK and also include the evaluation of the real-world clinical effectiveness of the programme.
- The study will use Normalisation Process Theory as a theoretical framework to guide data collection and interpretation.
- The qualitative findings will inform the development of an implementation manual for policy-makers, planners, providers and commissioners of cardiac rehabilitation services for heart failure patients.
- A possible limitation of the study is that the four centres that will be appointed to implement REACH-HF are large, well-established cardiac rehabilitation treatment

For peer review only

centres and might not be representative of the national cardiac rehabilitation landscape.

- This study may have limited generalisability outside the UK.

Study registration: not applicable.

For peer review only

INTRODUCTION

Heart failure

Approximately 900,000 people are affected by heart failure (HF) in the United Kingdom (UK).[1] Due to an ageing population, HF is becoming a national healthcare challenge.[2] HF has a high impact on both patients and society; it can reduce exercise tolerance and health-related quality of life (HRQoL), increase the risk of mortality and unplanned hospital admissions and is associated with high healthcare costs.[3] There is also a considerable burden on the friends and family of people with HF.[4] Exercise-based cardiac rehabilitation (CR) programmes have been shown to enhance HRQoL in patients with HF and reduce unplanned hospital admissions.[3,5] With sufficient adherence, these benefits are consistently achieved in trial settings with both centre- and home-based CR.[3] Although the National Institute of Health and Care Excellence (NICE) recommends that all patients with HF receive CR,[6] due to the frailty and poor health of this clinical population, as well as dislike of group-based exercise and practical constrains (e.g. transportation), participation in centre-based CR remains poor.[7] Underutilisation of CR amongst this clinical population has been highlighted in the 2010 NICE guideline, with the uptake of CR being much lower than predicted and estimated at 5.3%.[8]

REACH-HF

The Rehabilitation EnAblement for CHronic Heart Failure (REACH-HF) programme is a new CR programme for HF patients and their caregivers, aimed at achieving better HRQoL in the comfort of the patient's home. The 12-week, facilitated, home-based intervention was co-developed with patients, caregivers and clinicians,[9] using Intervention Mapping approach.[10] In recent randomised controlled trials (RCTs), REACH-HF resulted in significant clinical improvements in HRQoL and was cost-effective, with a cost falling within the current National Health Service (NHS) tariff for CR in the UK.[11,12] REACH-HF therefore provides an affordable, evidence-based, patient-centred alternative to centre-based CR. This provides a way to address the latest NICE guidance recommendation that HF patients are offered "a personalised, exercise-based cardiac rehabilitation programme in a format and setting (at home, in the community or in the hospital) that is easily accessible for the person".[6]

Implementation science: negotiating the research-to-practice gap

Research and development within the NHS is world leading. However, the NHS falls short when scaling up well-evidenced innovations or good practice.[14] The spread of innovations and evidence-based interventions across the NHS and other health care systems is subject to various challenges.[15] Firstly, moving complex interventions from research settings to real-world clinical implementation is a slow process.[13] Some of the barriers slowing down this process include the characteristics of the intervention itself such as its usability or fit with the existing processes in the organisation. Beyond this, individual or organisational barriers include the attitudes towards change and the innovation itself, resources available, expertise, time and competing priorities.[16]

Secondly, following uptake, the same intervention does not always perform in exactly the same way across different organisations. For example, there may be differences in the characteristics of the people involved. In clinical trials patients tend to be included based on predetermined criteria and

For peer review only

such criteria are rigorously checked prior to study participation. However, in practice a broader patient population may end up using the intervention. There may also be differences in the characteristics of the organisations delivering the intervention in terms of access to resources, staff and expertise, compared with those available in clinical trials. With these differences in population characteristics and access to resources, unplanned adaptations may occur to better fit the new context. This initially slows down the process of implementation, but also means that the intervention is no longer delivered as it was under clinical trial conditions.[17] Such unplanned adaptations often result in the interventions initially failing to reproduce the results that are found within the context of RCTs.[18] With a varied and ever changing healthcare landscape, it is crucial to understand the full complexity of implementing innovations into real-world clinical practice.[19] It is particularly important to explore how much of the intervention can or cannot change (and in what ways) without jeopardising the benefits of the intervention.[20]

Healthcare evaluations and improvement projects often consider performance at the level of the individual healthcare professional,[21] targeting the professional's knowledge, routines and attitudes.[22] However, there is a need for wider-reaching systems-level evaluations of the implementation process that also take into account community, organisational, system and policy level influences.[23]

Overall, implementation science aims to examine the process of implementation of healthcare innovations, in particular, the barriers and facilitators, as observed in real-life clinical settings.[24] To narrow the research-to-practice gap, implementation scientists recommend that the process of implementation is considered and built into the intervention design and development, the context and systems of implementation are assessed during the implementation efforts and key stakeholders are involved in the intervention development stage through to dissemination, implementation and evaluation.[23]

Aims of the project

The current project aims to implement REACH-HF in four UK NHS CR services to a) explore the facilitators of, and barriers to, implementation of REACH-HF in existing UK CR services, b) assess the implementation fidelity and c) the extent and nature of any potential adaptations to the intervention content and how such adaptations impact on effectiveness, and c) compare real-world outcomes to the clinical trial findings.

METHODS AND ANALYSIS

Design

We will conduct a mixed-methods implementation evaluation study using in-depth semi-structured interviews with key NHS staff, analysis of pre-post intervention changes in routinely collected outcome data via the British Heart Foundation founded National Audit of Cardiac Rehabilitation (NACR) and a fidelity assessment using a checklist applied to recordings of provider-patient interactions.

For peer review only

Setting and Site Recruitment

The study will be conducted in four, UK NHS CR centres who will be early adopters of the REACH-HF programme and known as 'Beacon Sites'. The opportunity to apply to become a Beacon Site will be promoted at national (UK) conferences and local meetings of CR practitioners. Interested CR services will be sent an information pack including an application form. Applicants will be asked to provide information on their NACR registration status, number of referrals made to the CR service (for both cardiac patients and patients with a primary diagnosis of HF), whether the service is offering home-based programme, length of current programmes, number of programme completions, number of pre- and post-treatment assessment completions, as well as to comment on willingness to engage in research and host site visits for other interested parties.

The sites will be recruited from across the UK using a two-stage application process (application form followed by panel interview for shortlisted sites). As an incentive, sites will be offered free intervention materials for the treatment of 50 patients (i.e., the REACH-HF patient manual, the Family & Friends Resource, audio with relaxation techniques and chair-based exercise DVD). In addition, the selected sites will be offered free training (including training manuals) for three health professionals to deliver REACH-HF, post-training support and formative feedback on performance. The three-day training will be delivered by the Heart Manual Department (HMD), NHS Lothian in Edinburgh.

To be eligible, sites have to be:

- NACR electronically registered sites with high quality status from the past audit period (green or amber status) operating in England, Wales or Northern Ireland. The NACR assesses CR teams against seven Key Performance Indicators (KPIs). A site assigned a green NACR status meets all seven KPIs, amber sites meet four to six KPIs and red sites meet one to three KPIs.
- Committed to delivering REACH-HF to 50 patients over the 12-month Beacon Site project period.
- Able to release three healthcare professionals (or more) with relevant experience in CR and/or HF for three days training plus one self-directed pre-training day.
- Able to engage in research to evaluate performance (i.e., recording some intervention sessions, staff participation in interviews).
- Willing to host site visits and/or share information and/or experiences with other interested NHS parties.
- Conduct baseline and post-treatment assessment of HRQoL using the Minnesota Living with Heart Failure Questionnaire (MLHFQ)[25] and exercise capacity using the Incremental Shuttle Walk Test (ISWT)[26] for all patients receiving the REACH-HF programme.

Study population

Healthcare providers: We aim to recruit up to 24 healthcare professionals. The total number will include the 12 health professionals delivering REACH-HF and other key NHS staff involved in the delivery, planning, and commissioning of CR for patients with HF. To identify key staff involved in CR services, the study will use a combination of opportunity sampling (all available staff trained to

For peer review only

deliver the REACH-HF programme) and snowball sampling (staff who are identified by existing participants as having a key role in delivering or commissioning of CR).[27] This sampling strategy will be applied until saturation in the themes and concepts generated in the qualitative analysis is reached.

Patients: The study will include up to 200 patients with HF who are referred to the CR centres for rehabilitation and receive REACH-HF treatment. Out of the 200 patients, CR consultations of up to 48 patients (12 per site) receiving REACH-HF intervention will be audio-recorded.

Intervention

REACH-HF is a home-based, health professional facilitated, 12-week CR programme supporting self-care in patients with HF, which has been co-developed with patients, caregivers and clinicians. The programme is described in detail elsewhere,[10-12,28-31] and is summarised below.

The programme consists of:

- The Heart Failure Manual for the patient provides information about HF to increase understanding of the condition and address common misconceptions, information about and strategies for managing the condition, and further information related to HF, such as lifestyle risk management, managing depression and anxiety, and getting support from others.
- A choice of two exercise training programmes; a chair-based programme (available on DVD and online) and a walking programme. Patients are recommended to engage in exercise three times per week, in addition to general physical activity.
- A stress management programme, with relaxation techniques, provided in the manual and in audio format, to help cope with anxiety and depression.
- A Progress Tracker designed for the patient to facilitate learning from experience through self-monitoring of behaviour and symptoms – prompting help-seeking, where necessary.
- A Family and Friends Resource to increase caregiver understanding of the condition, to enable them to support the patient in their self-care, and to help them address their wellbeing.
- Face-to-face and telephone facilitation over 12 weeks by a health professional trained to deliver the REACH-HF programme.

Facilitator Training

Three health professionals with CR and/or HF experience from each Beacon Site will attend a three day training course delivered by the HMD in Edinburgh. This training course will focus on the 7-steps of successful facilitation of REACH-HF (Fig 1) and include sessions on psychology, behaviour change, physical activity and exercise, engaging the caregiver, and further content/interaction designed to bring all of the components together.

Figure 1. The 7-steps of successful REACH-HF facilitation.

For peer review only

The Beacon Sites will determine which members of the CR team will attend the REACH-HF training. The main requirement for the healthcare professional is experience of delivering CR and/or of working with HF patients. The facilitators will likely be HF/cardiac specialist nurses, or physiotherapists/exercise specialists with qualifications and/or experience in the delivery of exercise-based CR programmes.

Measures and Procedures

Qualitative interviews

In-depth semi-structured interviews and focus groups with NHS staff (REACH-HF staff, managers, clinical leads and commissioners) will take place at each Beacon Site (See Appendix 1 for the Topic Guide). Each identified staff member will, if possible, be interviewed twice (at the beginning and end of the data collection window) and one focus group will be held in each locality with identified study participants (at the midpoint of the data collection window). Interviews will be either face-to-face or by phone. The development of topic guides for qualitative interviews and focus groups was based on four constructs and 16 sub-domains from the Normalisation Process Theory (NPT) framework (Table 1). The topic guides content may be amended depending on feedback from stakeholders and the first few interviews.

NPT construct	Construct's components	Interview questions
Coherence (sense-making)	Differentiation	Can you describe REACH-HF intervention and how it differs from your usual way of working?
	Communal specification	What is your colleagues understanding of the purpose of REACH-HF intervention?
	Individual specification	How does the intervention affect the nature of your work?
	Internalisation	In your opinion, what it the value of REACH-HF intervention? To you? To your patients?
Cognitive participation (relational work)	Initiation	Who are the individuals (you can include yourself) that drive REACH-HF forward and get others involved? What are their roles? What are they doing to support the project?
	Enrolment	How did the team need to change in order to introduce REACH-HF?
	Legitimation	How do you feel about being involved in the REACH-HF project?
	Activation	What is the future or REACH-HF in your service? What factors can enable the integration of REACH-HF into a cardiac rehabilitation service?
Collective action (operational work)	Interactional workability	How easy or difficult has it been to integrate REACH-HF into your existing work?
	Relational integration	How has implementing REACH-HF affected working relationships within the team?
	Skills and workability	How do the skills of the staff delivering REACH-HF match the needs of the programme?
	Contextual integration	Was REACH-HF training sufficient to allow for successful implementation? If not, what other topics or skills could have been included?

For peer review only

		Are there enough resources available to support the REACH-HF programme?<br>Are there any other barriers to delivering REACH-HF on your patch?
Reflexive monitoring (appraisal work)	Systematisation	Are you in any way evaluating effectiveness, usefulness or impact of REACH-HF on the service?
	Communal appraisal	Do your colleagues consider the intervention worthwhile?
	Individual appraisal	Do you consider it worthwhile?
	Reconfiguration	Can REACH-HF intervention be easily modified and improved to suit your way of working? If yes, in what way?

Table 1. Qualitative questions and their origins in the NPT construct and components.

Two video-conferencing peer supervision sessions will be available to all REACH-HF trained facilitators, provided by the HMD, as part of the REACH-HF training package. The researchers will observe, and take notes from each of these sessions.

Fidelity assessment

All REACH-HF CR treatment sessions (4-6 contacts), both face to face and phone-based, of approximately 48 consenting patients (12 per site), will be audio recorded by the healthcare professionals delivering the programme. Each REACH-HF facilitator will be requested to audio record all treatment sessions for four HF patients. The selection of which patients to include will be guided by the researchers, using a quasi-random process. Five months after the REACH-HF training, facilitators will be asked to invite all subsequent patients to take part in the study, until two willing HF patients agree to have their treatment sessions recorded. Approximately ten months after the REACH-HF training, an e-mail will be sent to repeat the invitation and audio recording process for the next two consenting patients.

The quality of delivery (intervention fidelity) of the recorded treatments will be assessed by the researcher (PD) using the same fidelity checklist used in the original REACH-HF research study.[11] This will allow comparison with fidelity scores achieved in the clinical trial. The recordings for the first six patients will also be double scored and two researchers (PD and CG) will discuss any differences in their scores to agree and 'anchor' the scoring process and minimise coder bias. If an agreement cannot be reached, a third reviewer (JVZ) will be appointed for arbitration.

The fidelity checklist is a 12-item checklist focused on identifying key delivery processes, such as the use of a patient-centred communication style, making a plan of action and encouraging self-monitoring of progress (particularly with the exercise programme). The checklist uses the Dreyfus scale of clinical skills acquisition,[32] to rate clinical skills on a scale of 0-6 and is anchored such that a score of three or more represents adequate delivery quality for each item. Coding instructions are provided (Appendix 2).

REACH-HF facilitators will be asked to complete a brief self-rated fidelity checklist after each session they have recorded. This comprises questions about the same 12 main components of the treatment and allows the facilitators to rate the occurrences of each feature (absence, minimal, some,

For peer review only

sufficient, good, very good, excellent) (Appendix 3). The main reason for including a self-rated fidelity checklist is that an independent observer-rating is time-consuming/labour intensive, whereas a self-rating assessment might provide a pragmatic, lower-cost alternative for checking delivery quality for use in real-world clinical practice.

Lastly, for each patient opting into the study, age, sex, time since diagnosis and severity of symptoms will be recorded by the healthcare professionals delivering the REACH-HF intervention.

Quantitative

At the end of the Beacon Site project period, a report will be requested from the NACR team based at the University of York on:

- number of referrals made to the Beacon Sites during the study period,
- number of HF patients enrolled on the REACH-HF programme (attending at least one session),
- CR attendance (average number of face-to-face and telephone sessions per patient),
- number of patients completing the REACH-HF programme (defined as attending the initial assessment and at least two contacts thereafter, of which one must be face to face).

Summary data on key pre- and post-programme measures will also be requested to enable comparison with changes in the intervention group observed in the clinical trial. These include HRQoL – determined using the MLHFQ and exercise capacity – determined using the ISWT. The MLHFQ consist of 21 questions that rate on a scale of 0-5 (where 0 is not at all, 1 is very little and 5 is very much) how different HF symptoms (i.e., swelling of ankles and legs, shortness of breath or tiredness, fatigue and poor energy levels) prevent the patient from living as they would have wanted to during the four week period prior to the first CR session. ISWT is an externally paced exercise capacity test that can be administered in the field with minimal equipment and without medical supervision. The test has good test-retest reliability and it is an acceptable alternative to (widely used to assess physical fitness and functional capacity of cardiac patients) exercise test with electrocardiogram monitoring or the cardiopulmonary exercise test.[33] A recent study confirmed that a single ISWT is a valid, low resource, assessment of an estimate for physical fitness and functional capacity for CR patients.[34]

Data Analysis

Qualitative data

Digital recordings of interviews and focus groups will be transcribed verbatim and any potentially identifiable information, such as individual or location names, will be redacted. The transcripts (Word documents) will be uploaded into NVivo software to help organise the data for analysis.[35] Illustrative quotes, that may be used in future presentations or publications, will be presented alongside pseudonyms to protect anonymity.

The transcripts will be analysed according to the principles of framework analysis outlined by Ritchie and Spencer,[36] and using the four over-arching constructs of NPT (coherence, cognitive

For peer review only

participation, collective action and reflexive monitoring) as an initial framework for coding the data.[37] NPT suggests general mechanisms that are associated with successful implementation. These include service providers' understanding of the new intervention and how it differs from standard practice, their motivation and attitude toward the healthcare innovation and the work they do to deliver and evaluate the intervention. NPT will provide a framework for generating questions for interviews and focus groups and analysing gathered data. See Table 1 for more details on the application of NPT to the data collection.

Fidelity assessment

Implementation fidelity scores from the fidelity checklist will be collated at the level of the facilitator, the site and the total sample, and presented using descriptive statistics (means, ranges) using the same analytic approach as the original REACH-HF trial.[11] Numerical data (0-6) from the Dreyfus scale of clinical skills acquisition will be converted into categorical (yes/no) data reflecting whether the session reflected the adequate level of delivery (score three or above). Observer-rated treatment fidelity will be compared with self-rated fidelity from the post-session fidelity questionnaires completed by the REACH-HF facilitators at the end of each recorded session. The analytic approach to compare the two rating scales will be Pearson's correlation for continuous scores,[38] and Gwet's first-order agreement coefficient (the AC1 statistic) for categorical ratings.[39]

The fidelity assessment data sample reflects the sample size used to assess fidelity in the original REACH-HF clinical trial. We require a minimum of four patient recordings per facilitator to be able to assess variation in performance between staff and between NHS sites.

Quantitative outcomes

Pre- and post-treatment changes in outcome data (MLHFQ and ISWT) will be reported as changes in mean scores with 95 % confidence intervals. Mean change scores for patients receiving REACH-HF will be compared (descriptively) with the changes found in the REACH-HF trial. Similarly, change scores for patients receiving REACH-HF will be compared with an aggregate change score from the NACR database for those who receive other forms of CR (primarily centre-based or digital CR). Sub-group analyses will be conducted (if possible) by the NACR team to determine variations in uptake and outcomes within our REACH-HF cohort by site, sex, and other characteristics of interest (e.g. area deprivation index, rurality). Data on the number of patients treated, uptake and completion rates and session attendance, will be presented using descriptive statistics. Figure 2 illustrates interactions between the study's aims and methods, and how they link with the process of ongoing evaluation and scale-up.

Figure 2. Beacon Site evaluation and embedded processes for ongoing monitoring.

Patient and public involvement

Patient preference and acceptability have been addressed extensively during the REACH-HF clinical trials.[11,12] Six patients with HF and four caregivers have been consulted and informed the design of the REACH-HF programme. Patient and public involvement in the proposed study has included involving a member of the public to read and comment on the content of the study invite letter, participant information sheet and the consent form designed for the study. Additionally, members

For peer review only - http://bmjopen.bmj.com/site/about/guidelines.xhtml

of all CR teams involved in the study were consulted during the process of setting up the Beacon Sites on issues such as the feasibility of the study, selected outcome measures and the burden of participation in the study. At the end of the study, the final report will be shared with NHS staff at the participating Beacon Sites, allowing them to use it for service evaluation, future service planning and sharing of good practice.

Discussion

The research-to-practice translation gap is well documented. It is common that evidence-based interventions are not adopted into clinical settings and do not become routine practice. To narrow the translation gap, more insight is needed into mechanisms that allow for successful implementation of effective and cost-effective interventions. To advance the field, implementation theories and mechanisms need to be tested in real-world clinical settings.

The REACH-HF Beacon Site project is a multi-faceted and interactive approach to a phased roll-out that aims to disseminate the multi-centre trial findings, increase awareness of the REACH-HF intervention and to explore replicability of the intervention in new contexts. In line with earlier recommendations for implementation research, this study will open a channel of feedback between researchers and implementers (NHS staff), with a common goal of improved service delivery for HF patients. This study will provide an insight into the translation of the REACH-HF clinical trial findings into real-world practice and an in-depth understanding of the implementation process in the context of current NHS provision. These findings will inform the future, larger-scale implementation of REACH-HF, offer guidance to policy-makers, planners and commissioners of CR services, inform adaptations to the REACH-HF training package and intervention and facilitate adoption and spread of home-based CR for HF patients in the UK.

ETHICS AND DISSEMINATION

Before the start of the study, favourable opinion has been sought from the NHS Reach Ethics Committee (REC) and the Health Research Authority (HRA) for all the activities outlined in the study protocol (IRAS 261723). Written informed consent will be obtained from all health professionals and patients participating in the study.

The research team will ensure that the study is conducted in accordance with the Declaration of Helsinki,[40] the Data Protection Act 2018,[41] General Data Protection Regulations,[42] and in accordance with the Research Governance Framework for Health and Social Care (2005).[43]

Findings will be published in scientific peer-reviewed journals and presented at local, national and international meetings to publicise and explain the research methods and findings to key audiences to facilitate the further uptake of the REACH-HF intervention.

AUTHOR CONTRIBUTIONS: All authors contributed to the idea for the study. PD and SvB drafted the manuscript. SvB lead the set up and recruitment of Beacon Sites. SMcD is overseeing the day-to-day management of the Beacon Site project. PD secured all relevant ethical approvals for the project and prepared all study documentation. CG, JVZ, HD and RT are providing project supervision and oversight. PJD and AH will coordinate access to the NACR data. AH provided statistical analysis advice. PD will acquire and analyse the data for the study. All authors provided critical revision of the

For peer review only - http://bmjopen.bmj.com/site/about/guidelines.xhtml

manuscript for important intellectual content and approved the final draft of the protocol for submission.

FUNDING STATEMENT: This study/project was funded by the National Institute for Health Research (NIHR) [Programme Grants for Applied Research scheme (project reference RP-PG-1210-12004)]. The views expressed are those of the author(s) and not necessarily those of the NIHR or the Department of Health and Social Care.

PD's time is funded by a PhD studentship from the University of Birmingham.

COMPETING INTERESTS STATEMENT: None

REFERENCES:

1 The British Heart Foundation. Facts and Figures 2019 [available from: https://www.bhf.org.uk/what-we-do/news-from-the-bhf/contact-the-press-office/facts-and-figures, date accessed: November 2019].

2 The National Health Service (NHS). Cardiovascular disease (CVD) 2019 [available from: https://www.england.nhs.uk/ourwork/clinical-policy/cvd/, date accessed: November 2019].

3 Long L, Mordi IR, Bridges C, et al. Exercise-based cardiac rehabilitation for adults with heart failure. *Cochrane Database Syst Rev* 2019;1:cd003331.

4 Wingham J, Frost J, Britten N, et al. Needs of caregivers in heart failure management: A qualitative study. *Chronic Illn* 2015;11(4):304-19.

5 Anderson L, Taylor RS, Cardiac rehabilitation for people with heart disease: an overview of Cochrane systematic reviews. *Cochrane Database Syst Rev* 2014;12:cd011273.

6 National Institute for Health and Care Excellence. Chronic heart failure in adults: diagnosis and management (NICE Guideline 106) 2018 [available from: https://www.nice.org.uk/guidance/ng106, date accessed: November 2019].

7 National Audit of Cardiac Rehabilitation. National Audit of Cardiac Rehabilitation (NACR) Quality and Outcomes Report 2018 [available from: https://www.bhf.org.uk/informationsupport/publications/statistics/national-audit-of-cardiac-rehabilitation-quality-and-outcomes-report-2018, date accessed: November 2019].

8 National Institute for Health and Care Excellence (NICE). Chronic heart failure in adults: management (NICE Clinical Guideline CG108) 2010 [available from: https://www.nice.org.uk/guidance/cg108, date accessed: November 2019].

9 Greaves CJ, Wingham J, Deighan C, et al. Optimising self-care support for people with heart failure and their caregivers: development of the Rehabilitation Enablement in Chronic Heart Failure (REACH-HF) intervention using intervention mapping. *Pilot Feasibility Stud* 2016;2:37.

10 Bartholomew Eldredge LK, Markham CM, Ruiter RAC, et al. Planning Health Promotion Programs: An Intervention Mapping Approach. Hoboken: John Wiley & Sons 2016.

For peer review only

11 Dalal HM, Taylor RS, Jolly K, et al. The effects and costs of home-based rehabilitation for heart failure with reduced ejection fraction: The REACH-HF multicentre randomized controlled trial. *Eur J Prev Cardio* 2018;26(3):262-72.

12 Lang CC, Smith K, Wingham J, et al. A randomised controlled trial of a facilitated home-based rehabilitation intervention in patients with heart failure with preserved ejection fraction and their caregivers: the REACH-HFpEF Pilot Study. *BMJ Open* 2018;8(4):e019649.

13 Cooksey DA. Review of UK health research funding. London: HM Treasury 2006 [available from: https://assets.publishing.service.gov.uk/government/uploads/system/uploads/attachment_data/file/228984/0118404881.pdf, date accessed: November 2019].

14 Castle-Clarke S, Edwards N, Buckingham H. Falling short: Why the NHS is still struggling to make the most of new innovations. Nuffieldtrust Briefing 2017 [available from: https://www.nuffieldtrust.org.uk/research/falling-short-why-the-nhs-is-still-struggling-to-make-the-most-of-new-innovations, date accessed: November 2019].

15 Horton T, Illingworth J, Warburton W. The spread challenge: How to support the successful uptake of innovations and improvements in health care. London: Health Foundation 2018 [available from: https://www.health.org.uk/publications/the-spread-challenge, date accessed: November 2019].

16 De Silva D. What's getting in the way? Barriers to Improvement in the NHS. The Health Foundation: evidence scan 2015 [available from: https://www.health.org.uk/publications/what%E2%80%99s-getting-in-the-way-barriers-to-improvement-in-the-nhs, date accessed: November 2019].

17 Bell A, Corfield M, Davies J, et al. Collaborative transdisciplinary intervention in early years - putting theory into practice. *Child Care Health Dev* 2010;36(1):142–8.

18 Fixsen DL, Naoom SF, Blase KA, et al. Implementation Research: A Synthesis of the Literature. Tampa, FL: University of South Florida, Louis de la Parte Florida Mental Health Institute 2005.

19 Escoffery C, Lebow-Skelley E, Haardoerfer R, et al. A systematic review of adaptations of evidence-based public health interventions globally. *Implement Sci* 2018;13(1):125.

20 Breitenstein SM, Gross D, Garvey CA, et al. Implementation fidelity in community-based interventions. *Res Nurs Health* 2010;33(2):164-73.

21 Grol RPTM, Bosch MC, Hulscher MEJL, et al. Planning and Studying Improvement in Patient Care: The Use of Theoretical Perspectives. *Milbank Q* 2007;85(1):93-138.

22 Grimshaw JM, Thomas RE, MacLennan G, et al. Effectiveness and efficiency of guideline dissemination and implementation strategies. *Health Technol Assess* 2004;8(6):iii-iv,1-72.

23 Greenhalgh T, Robert G, Macfarlane F, et al. Diffusion of Innovations in Service Organizations: Systematic Review and Recommendations. *Milbank Q* 2004;82(4):581-629.

For peer review only

24 Schliep ME, Alonzo CN, Morris MA. Beyond RCTs: Innovations in research design and methods to advance implementation science. *Evid Based Commun Assess Interv* 2018;11(3-4):82-98.

25 Bilbao A, Escobar A, García-Perez L, et al. The Minnesota living with heart failure questionnaire: comparison of different factor structures. *Health Qual Life Outcomes* 2016;14:23.

26 Singh SJ, Morgan MDL, Scott S, et al. Development of a shuttle walking test of disability in patients with chronic airways obstruction. *Thorax* 1992;47(12):1019-24.

27 Marshall MN. Sampling for qualitative research. *Fam Pract* 1996;13(6):522-526.

28 Taylor RS, Hayward C, Eyre V, et al. Clinical effectiveness and cost-effectiveness of the Rehabilitation Enablement in Chronic Heart Failure (REACH-HF) facilitated self-care rehabilitation intervention in heart failure patients and caregivers: rationale and protocol for a multicentre randomised controlled trial. *BMJ Open* 2015;5(12):e009994.

29 Taylor RS, Sadler S, Dalal HM, et al. The cost effectiveness of REACH-HF and home-based cardiac rehabilitation compared with the usual medical care for heart failure with reduced ejection fraction: A decision model-based analysis. *Eur J Prev Cardiol* 2019;26(12):1252-1261.

30 Lang CC, Smith K, Wingham J, et al. A randomised controlled trial of a facilitated home-based rehabilitation intervention in patients with heart failure with preserved ejection fraction and their caregivers: the REACH-HFpEF Pilot Study. *BMJ Open* 2018;8(4):e019649.

31 Frost J, Wingham J, Britten N, et al. Home-based rehabilitation for heart failure with reduced ejection fraction: mixed methods process evaluation of the REACH-HF multicentre randomised controlled trial. *BMJ Open* 2019;9(8):e026039.

32 Dreyfus HL. The Dreyfus model of skill acquisition. In: J. Burke, eds. Competency based education and training. London: Falmer Press 1989.

33 Hanson LC, Taylor NF, McBurney H. The 10m incremental shuttle walk test is a highly reliable field exercise test for patients referred to cardiac rehabilitation: a retest reliability study. *Physiotherapy* 2016;102(3):243-8.

34 Hanson LC, McBurney H, Taylor NF. Is the 10 m incremental shuttle walk test a useful test of exercise capacity for patients referred to cardiac rehabilitation? *Eur J Cardiovasc Nurs* 2018;17(2):159-69.

35 Richards L. Handling qualitative data: a practical guide. Los Angeles: SAGE 2014.

36 Ritchie J, Spencer L. Qualitative data analysis for applied policy research by Jane Ritchie and Liz Spencer. In: Bryman A, Burgess RG, eds. Analysing qualitative data. London: Routledge 1994:173-194.

37 May CR, Cummings A, Girling M, et al. Using Normalization Process Theory in feasibility studies and process evaluations of complex healthcare interventions: a systematic review. *Implement Sci* 2018;13(1):80.

For peer review only

38 Mukaka MM. A guide to appropriate use of correlation coefficient in medical research. *Malawi Med J* 2012;24(3):69-71.

39 Gwet KL. Handbook of Inter-Rater Reliability: The Definitive Guide to Measuring the Extent of Agreement Among Raters. Gaithersburg: Advanced Analytics 2010.

40 World Medical Association. Declaration of Helsinki – Ethical Principles for Medical Research Involving Human Subjects. *JAMA* 2013;310(20):2191-2194.

41 Stationery Office. Data Protection Act 2018. London 2018 [available from: http://www.legislation.gov.uk/ukpga/2018/12/contents/enacted, date accessed: November 2019].

42 Information Commissioner's Office. General Data Protection Regulation (GDPR). London 2018 [available from: https://ico.org.uk/about-the-ico/what-we-do/legislation-we-cover/general-data-protection-regulation/, date accessed: November 2019].

43 Stationery Office. Research Governance Framework for Health and Social Care. London 2005 [available from: https://www.gov.uk/government/publications/research-governance-framework-for-health-and-social-care-second-edition, date accessed: November 2019].

For peer review only

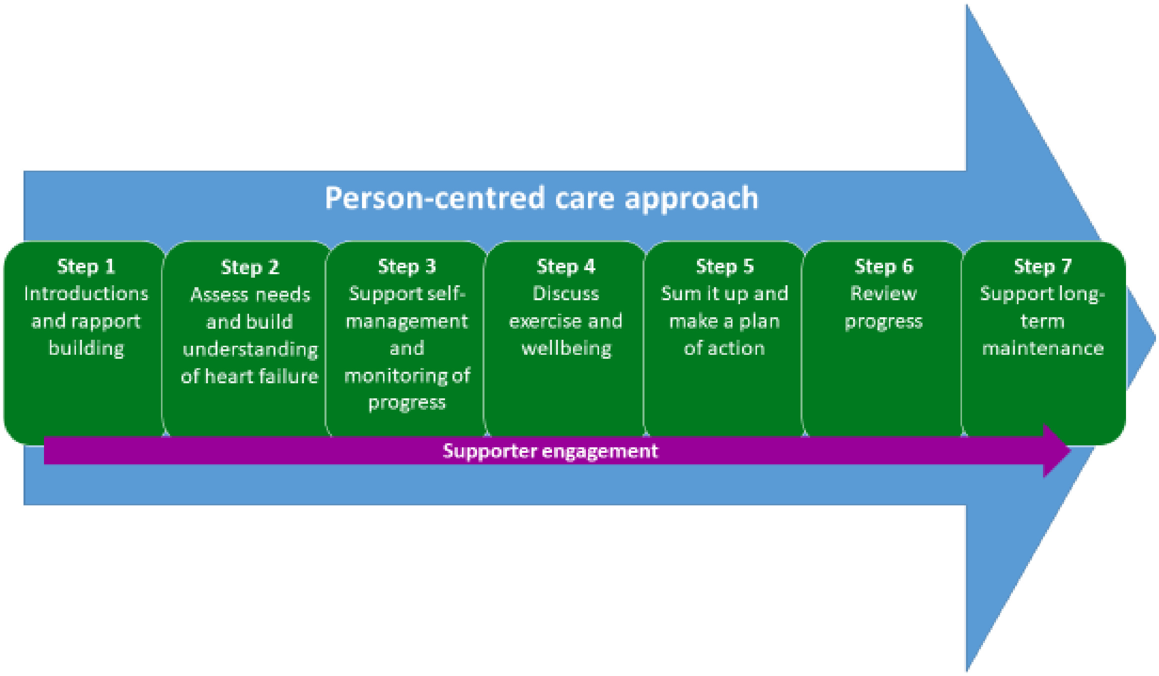

For peer review only

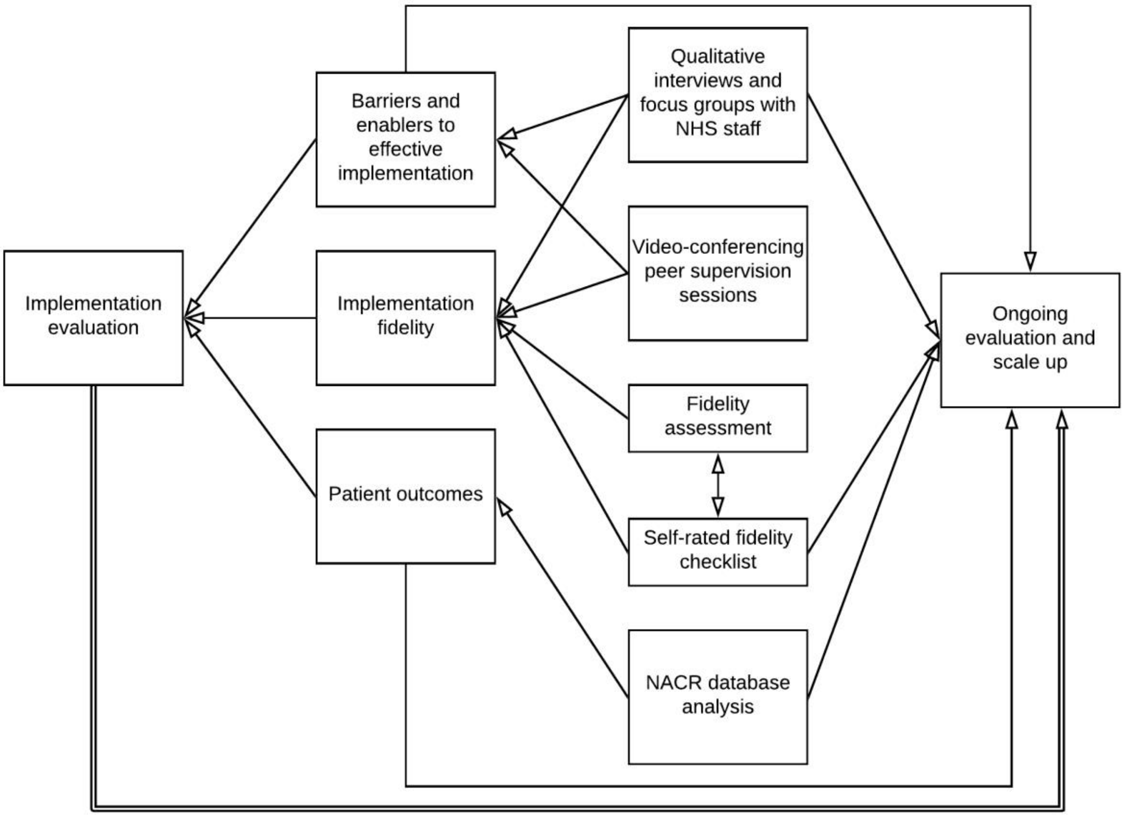
Implementation evaluation
Barriers and enablers to effective implementation
Implementation fidelity
Patient outcomes
Qualitative interviews and focus groups with NHS staff
Video-conferencing peer supervision sessions
Fidelity assessment
Self-rated fidelity checklist
NACR database analysis
Ongoing evaluation and scale up

eview only

What are the barriers and enablers to effective implementation of REACH-HF?

Qualitative interview guide (initial draft*)

** The topic guide content may vary depending on feedback from stakeholders and the first few interviews*

Beacon site: I / II / III / VI *(circle as appropriate)*

Date of interview: ____

- **Welcome and housekeeping**
  Thank you for agreeing to take part in the study. The interview will last between 30 and 40 minutes. I will ask you a series of questions and I am really interested in your honest opinion on the subject matter. If you wish to stop at any point to take a break, let me know.

- **Informed consent**
  Thank you for reading PIS and completing the consent form. Is it ok if we start recording?

- **Interview questions**

NPT	Questions	Comments
1.1	Can you describe REACH-HF intervention and how it differs from your usual way of working?	
1.3	How does the intervention affect the nature of your work?	
4.3	Do you consider it to be worthwhile?	
1.4	In your opinion what is the value of REACH-HF intervention? To you? To your patients?	
1.2	What is your colleagues understanding of the purpose of REACH-HF intervention?	
4.2	Do they consider it to be worthwhile?	
3.2	How has implementing REACH-HF affected working relationships within the team?	
2.1	Who are the individuals (you can include yourself) that drive REACH-HF forward and get others involved? What are their roles? What are they doing to support the project?	
3.1	How easy or difficult has it been to integrate REACH-HF into your existing work?	
2.2	How did the team need to change in order to introduce REACH-HF?	
2.3	How do you feel about being involved in the REACH-HF project?	
3.3	How do the skills of the staff delivering REACH-HF match the needs of the programme?	

For peer review only - http://bmjopen.bmj.com/site/about/guidelines.xhtml

What are the barriers and enablers to effective implementation of REACH-HF?

3.4	Was REACH-HF training sufficient to allow for successful implementation? If not, what other topics or skills could have been included?<br><br>Are there enough resources available to support the REACH-HF programme?<br><br>Are there any other **barriers** to delivering REACH-HF on your patch?	
4.1	Are you in any way evaluating effectiveness, usefulness or impact of REACH-HF on the service?	
4.4	Can REACH-HF intervention be modified and improved to suit your way of working? If yes, in what way?	
2.4	What is the future of REACH-HF in your service? What factors can **enable** integration of REACH-HF into a cardiac rehabilitation service?	

- ***A few: service-level questions:*** *What is the catchment area for your service? What population do you serve?*

- **Ending & debrief**
  Thank you for taking the time to answer my questions. Is there anything else you would like to add? Or ask me about? I am going to switch off the audio recorder now. If any of what we spoke about affected you in any way we can have a debrief session now.

For peer review only

For peer review only - http://bmjopen.bmj.com/site/about/guidelines.xhtml

REACH-HF FIDELITY MEASURE

The rating scale

The seven point scale extends from (0) where the facilitator did not deliver the intervention element appropriately - either they didn't do it well or didn't do it sufficiently (low fidelity) to (6) where there is the element is delivered appropriately (high fidelity). Thus the scale assesses a composite of both adherence to the intended intervention techniques and the skill of the facilitator in delivering the techniques. To aid with the rating of items, an outline of the key features of each item is provided at the top of each section. A generic description of the rating criteria is given in Figure 1.

Adjusting for the presence of patient difficulties

Adjustments may be needed when patient difficulties are evident (e.g. excessive avoidance or resistance). In such circumstances, the rater needs to assess the facilitator's therapeutic skills in the application of the methods. Even though the facilitator may not facilitate change, credit should be given for attempting to use the intended techniques and demonstrating appropriate /skilful interaction (i.e. they should do what they can, within reason, to deliver the intended intervention components).

Figure 1: The scoring system

Competence level* **Scoring** **Examples**

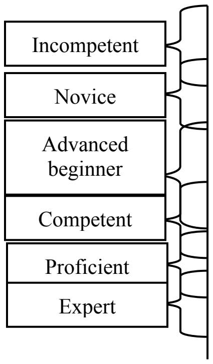

0 Absence of feature and /or highly inappropriate performance

1 Minimal use of feature and /or inappropriate performance,

2 Evidence of competence, but numerous problems

3 Competent, but some problems or inconsistencies

4 Good features, but minor problems or inconsistencies

5 Very good features, minimal problems or inconsistencies

6 Excellent performance

* The scale incorporates the Dreyfus system (Dreyfus, 1989) for denoting competence. Please note that the 'top marks' (i.e. near the 'expert' end of the continuum) are reserved for those facilitators demonstrating highly effective skills, particularly in the face of difficulties (i.e. patients with high resistance to change; high levels of emotional expression; and complex situational barriers). Please note that there are 5 competence levels but six potential scores.

When rating the item, you should first identify whether some of the 'Key Features' are present. If the facilitator includes most of the key features and uses them appropriately (i.e. misses few relevant opportunities to use them and delivers them well), the facilitator should be rated highly. It is important to remember that the scoring profile for this scale should approximate to a normal distribution, with relatively few people

For peer review only - http://bmjopen.bmj.com/site/about/guidelines.xhtml

scoring at the extremes. **For the purposes of the REACH study, a score of 3 or more will be taken to represent “acceptable delivery or basic competence” in using the intended techniques”**

Dreyfus, H. L. (1989). The Dreyfus model of skill acquisition. In J. Burke (ed.) Competency based education and training. London: Falmer Press.

For peer review only

For peer review only - http://bmjopen.bmj.com/site/about/guidelines.xhtml

ITEM 1: ACTIVE PATIENT INVOLVEMENT

Key features: The facilitator should encourage the participant to be actively involved in the consultation. The idea is to maximise the participant's autonomy as the main agent of change, developing intrinsic rather than extrinsic motivation, and encouraging her /him to be the person coming up with ideas for improving the situation. However, the participant should not be allowed to ramble in an unstructured way and the consultation should be guided. A collaborative /shared decision-making style is appropriate and the facilitator may share his /her own expertise and ideas (as below). Overall, the participant should be increasingly empowered to take control of her /his self care behaviour. Interactions should be encouraging, respectful and non-judgemental (the opposite of a didactic, telling or persuading style of interaction). The participant should ideally talk for at least half of the time (particularly in later sessions). The interaction should also be *individually tailored* to the patient's specific information needs, beliefs, motivations and barriers. The facilitator should engender a clear sense of warmth, genuineness and empathy (within professional boundaries).

Intervention techniques: OARS (Open questions, Affirmation, Reflective listening, Summaries). Reflective listening may include simple reflections of content but may also be more sophisticated (e.g. amplified reflection; reflection with a twist) and used to direct the conversation or highlight key strengths or barriers. Summaries to reinforce patient choices and acknowledge patient effort are particularly desirable. Individual tailoring of techniques and responses to the individual patient's existing knowledge, skills, current activity levels, needs and preferences are also desirable. The Ask-Tell-Discuss technique should be used to exchange information (e.g. to address misconceptions, or offer helpful new information). The above empathy-building techniques and individual tailoring should be used throughout the consultations - from the initial consultation through action-planning through to review /maintenance sessions.

Mark with an 'X' on the vertical line, using whole and half numbers, the level to which you think the facilitator has delivered this intervention process

0 Absence of active patient involvement techniques. An overly 'directing', practitioner-led or 'lecturing' style of interaction, which may increase or sustain client's resistance.

1 Minimal patient involvement or use of active patient involvement techniques. The practitioner dominates the discussion.

2 Some use of patient involvement techniques, but not frequent enough. The practitioner sometimes dominates the discussion.

3 Appropriate and frequent use of patient involvement techniques. Teamwork evident, but some difficulties in content or method of delivery.

4 Appropriate and frequent use of patient involvement techniques. Minor problems evident (e.g. some reflection opportunities missed).

For peer review only - http://bmjopen.bmj.com/site/about/guidelines.xhtml

5 Highly appropriate and regular use of patient involvement techniques, facilitating shared understanding and decision making. Minimal problems.

6 Excellent / expert use of patient involvement techniques throughout all the consultation. A clear sense of collaborative alliance is developed.

For peer review only

For peer review only - http://bmjopen.bmj.com/site/about/guidelines.xhtml

ITEM 2: ASSESSING THE PATIENT'S CURRENT SITUATION AND NEEDS.

Key features: The facilitator should work with the participant to assess the patient's current situation. They should seek to identify ALL of the following over the first 1-2 sessions: Identify and discuss the most important issue currently for the patient, how well are they managing their fluids, how appropriately are they using medications, is there any obvious immediate clinical need, how much stress or anxiety do they have, how much physical activity are they doing, and what other concerns or questions they may have.

Intervention techniques: Facilitators will use patient-centred communication techniques (as above) which may include the Ask-Tell-Discuss and 'tell me three things' technique to explore the patient's current situation.

Mark with an 'X' on the vertical line, using whole and half numbers, the level to which you think the facilitator has delivered this intervention process

0 Absence (or very poor delivery) of discussions to assess the patient's current situation.

1 Minimal (or poorly delivered) discussions to assess the patient's current situation.

2 Some discussions to assess the patient's current situation, but may not be in sufficient depth or detail, or quality of delivery may be variable.

3 Several examples of discussion to assess the patient's current situation. However some difficulties evident (e.g. missed opportunities, not covering all the key topics, or talking at odds with the patient).

4 Several examples of discussion to assess the patient's current situation. Minor problems evident.

5 Highly appropriate and sufficient discussion to assess the patient's current situation. Minimal problems.

6 Excellent / expert use of discussion to assess the patient's current situation. No real problems.

For peer review only - http://bmjopen.bmj.com/site/about/guidelines.xhtml

For peer review only

ITEM 3: FORMULATING AN APPROPRIATE (INDIVIDUALISED) TREATMENT PLAN

Key features: The facilitator should work with the participant to formulate an appropriate treatment plan based on the patient's current situation. This should aim to address (as a minimum) ALL of the following over the twelve weeks of the programme: What is the most important issue currently for the patient, are they managing their fluids well, are they using medications appropriately, any clinical needs identified, how much stress or anxiety do they have, how much physical activity are they doing, and any other concerns or questions they may have. The treatment plan will be staged over time, aiming to work on a few topics initially and introducing other elements as the programme continues. It is best practice to summarise the treatment plan at the end of the session "what we have said today is …".

Intervention techniques: Facilitators will use patient-centred communication techniques (as above) to discuss and agree what issues to address first and what order to do things in. An element of guiding to ensure the inclusion of clinical priorities (e.g. medication issues, physical activity, psychological well-being) as well as patient priorities may be appropriate. The facilitator will advise the patient (and caregiver if appropriate) to read relevant sections of the manual ahead of their next meeting.

Mark with an 'X' on the vertical line, using whole and half numbers, the level to which you think the facilitator has delivered this intervention process

0 Absence (or very poor delivery) of discussion to formulate an appropriate treatment plan based on the patient's current situation.

1 Minimal (or poorly delivered) discussion to formulate an appropriate treatment plan based on the patient's current situation.

2 Some discussion to formulate an appropriate treatment plan based on the patient's current situation, but may not be in sufficient depth or detail, or quality of delivery may be variable (e.g. not covering all the key topics, or talking at odds with the patient).

3 Several examples of discussion to formulate an appropriate treatment plan based on the patient's current situation. However some difficulties may still be evident (e.g. missed opportunities, plan not summarised at the end of the visit).

4 Several examples of discussion to formulate an appropriate treatment plan based on the patient's current situation. Minor problems evident.

5 Highly appropriate and sufficient discussion to formulate an appropriate treatment plan based on the patient's current situation. Minimal problems.

6 Excellent / expert use of discussion to formulate an appropriate treatment plan based on the patient's current situation. No real problems.

For peer review only - http://bmjopen.bmj.com/site/about/guidelines.xhtml

ITEM 4: BUILD THE PATIENT'S UNDERSTANDING OF HEART FAILURE /MAKING A LINK BETWEEN SELF-CARE ACTIVITIES AND THEIR HEART FAILURE SYMPTOMS

Key features: Participants' ability to make sense of how HF works and how self-care behaviours might influence the course of the illness will be crucial for the success of the intervention as belief in the benefit of the suggested self-care activities will increase motivation to engage in them. The facilitator should elicit the patient's current understanding of heart failure and seek to build their 'illness model' in terms of understanding the Identity, Causes, Consequences, Cure /control options and Timeline[1] associated with the condition. This process may take several weeks and should be reinforced as the programme progresses.

Intervention techniques: Facilitators will provide the REACH-HF Manual, provide a brief overview of how the manual works and, after assessing the patient's individual needs and concerns (as above), they will identify some key sections for the patient to read before the next contact, specifically including the Understanding HF section. Facilitators will use patient-centred communication techniques (as above) to elicit and build understanding. This should include the use of the Ask-Tell-Discuss technique and reflective listening to reinforce elements of the patient's understanding that are factually correct or which predispose towards positive self-care behaviours. They should seek to reframe negative attitudes and exchange information (Ask-Tell-Discuss) to address any misconceptions or to fill any important gaps in understanding. The facilitator will advise the patient (and caregiver if appropriate) to read relevant sections of the manual (including the Understanding HF chapter) to build and reinforce understanding /to address misconceptions. The way HF works should be explicitly discussed and referred back to /reinforced at subsequent sessions when this reinforces perceived benefits of the proposed self-care behaviours.

Mark with an 'X' on the vertical line, using whole and half numbers, the level to which you think the facilitator has delivered this intervention process.

0 Absence (or very poor delivery) of any exploration or discussion of how HF works. Understanding of HF is assumed or not mentioned or discussed.

1 Minimal (or poor delivery of) exploration or discussion of how HF works.

2 Some exploration or discussion of the how HF works, but may not be in sufficient depth or detail, or quality of delivery may be variable (e.g. telling rather than Ask-Tell-Discuss) or understanding is not checked.

3 Appropriate exploration and discussion of how HF works. However, some difficulties may still be evident (e.g. moving on before understanding is fully established).

4 Appropriate exploration or discussion of how HF works, linking changes in symptoms or mood with changes in self-care behaviour. Minor problems evident (e.g. some inconsistencies).

For peer review only - http://bmjopen.bmj.com/site/about/guidelines.xhtml

5 Highly appropriate and sufficient exploration or discussion of how HF works, facilitating a clear understanding of the process and linking changes in symptoms and mood with changes in self-care behaviour. Minimal problems.

6 Excellent / expert exploration and discussion facilitating a clear understanding of how HF works and the reasons for change. No real problems.

1. Leventhal H, Nerenz DR, Steele DJ: Illness representations and coping with health threats. In: *Handbook of Psychology and Health*. Volume IV. Edited by Baum AE, et al. Hillsdale NJ: Lawrence Erlbaum; 1984: 219-67.

For peer review only

For peer review only - http://bmjopen.bmj.com/site/about/guidelines.xhtml

ITEM 5a: SUPPORTING SELF-MONITORING AND PROGRESS-TRACKING

Key features: The facilitator should agree a verbal plan of action for the following week(s) with the patient. and discuss the use of the progress-tracking tools in the HF Manual to keep track of progress and as a way of recording any problems in completing the activities and any benefits that might be associated with the planned activities.

Intervention techniques: The facilitator should encourage the participant to monitor /keep track of their activities using the progress-tracking tools in the HF Manual.

Mark with an 'X' on the vertical line, using whole and half numbers, the level to which you think the facilitator has delivered this intervention process

0 Absence (or very poor delivery) of encouragement of self-monitoring.

1 Minimal (or poorly delivered) encouragement of self-monitoring. Activities planned are not sustainable, or poorly specified.

2 Some encouragement of self-monitoring but lacking detail /patient involvement in the activity may be limited, or quality of delivery may be variable (e.g. telling rather than discussing).

3 Appropriate encouragement of self-monitoring. However, some difficulties evident (e.g. not explaining the rationale for using the tool as a basis for monitoring progress, sometimes providing rather than eliciting ideas).

4 Appropriate encouragement of self-monitoring. Minor problems evident (e.g. the plan is a bit less specific than it could be).

5 Highly appropriate encouragement of self-monitoring. The participant has a clear understanding of the plan for the week ahead and how to monitor progress. Minimal problems

6 Excellent / expert encouragement of self-monitoring. The participant has a clear and realistic understanding of how to monitor progress. No real problems.

For peer review only

For peer review only - http://bmjopen.bmj.com/site/about/guidelines.xhtml

ITEM 5b: REVIEWING PROGRESS AND PROBLEM-SOLVING

Key features: The facilitator should work with the participant to review progress with all planned changes and with achieving the targets set out in the action plan. The facilitator should celebrate and reinforce and reflect on any successes. The participant and facilitator should discuss any setbacks and the patient's plans should be revised.

Intervention techniques: The facilitator should reinforce any self-monitoring activity and any successes in behaviour change (by giving praise/ using Affirmation techniques). Reframing should be used to normalise setbacks and see them as an opportunity to learn from experience (trial and error) rather than as failures. Problem-solving should use OARS (Open questions, Affirmation, Reflective listening, Summaries) and information exchange (Ask-Tell-Discuss) techniques to identify barriers and explore ways to overcome them. Problem-solving may specifically focus on issues of connectedness (social influences, involvement of others in supporting activities) and sustainability, or on breaking the problem down into more manageable chunks. Goals /action plans should be reviewed and revised if necessary.

Mark with an 'X' on the vertical line, using whole and half numbers, the level to which you think the facilitator has delivered this intervention process

0 Absence (or very poor delivery) of any progress review. No reinforcement of success and discussion of setbacks or barriers in relation to the previous weeks planned activities /problem-solving, or reviewing action plans.

1 Minimal (or poor delivery) of progress review. Minimal reinforcement of success and discussion of setbacks or barriers in relation to the previous weeks planned activities /problem-solving, or reviewing action plans.

2 Some progress review. Some reinforcement of success and discussion of setbacks or barriers in relation to the previous weeks planned activities /problem-solving and reviewing action plans, but lacking sufficient depth or detail or may be poorly delivered (e.g. providing solutions rather than using Ask-Tell-Discuss).

3 Appropriate progress review. Appropriate reinforcement of success and discussion of setbacks or barriers in relation to the previous weeks planned activities /problem-solving, and reviewing action plans. However, some difficulties evident (e.g. not reframing setbacks, not attempting to identify problems, or possible solutions).

4 Appropriate progress review. Appropriate reinforcement of success and discussion of setbacks or barriers in relation to the previous weeks planned activities /problem-solving, and reviewing action plans. Minor problems evident.

5 Highly appropriate and sufficient progress review. Appropriate reinforcement of success and discussion of setbacks or barriers in relation to the previous weeks planned activities /problem-solving, or reviewing action plans. Minimal problems.

For peer review only - http://bmjopen.bmj.com/site/about/guidelines.xhtml

6 Excellent / expert progress review. Excellent reinforcement of success and discussion of setbacks or barriers in relation to the previous weeks planned activities /problem-solving, and reviewing action plans. No real problems.

For peer review only

For peer review only - http://bmjopen.bmj.com/site/about/guidelines.xhtml

ITEM 6: MAKE A SPECIFIC ACTION PLAN FOR PHYSICAL ACTIVITY, BASED ON THE ACTIVITIES SELECTED BY THE PATIENT

Key features: Using the template in the HF manual, the facilitator should work with the participant to agree a written or verbal plan of action for engaging in one of the physical activity /exercise options over the following week(s). This should include discussion to ensure an appropriate intensity (moderate) of any activity included in the action plan.

Intervention techniques: Making a written action plan, using the planning tool in the manual, or a verbal action plan for physical activity. The facilitator should ensure that goal-setting is realistic. The facilitator may also employ some problem-solving techniques at this stage to pre-empt and address potential problems. It is best practice to summarise the plan at the end of the session "what we have said today is …".

Mark with an 'X' on the vertical line, using whole and half numbers, the level to which you think the facilitator has delivered this intervention process

0 Absence (or very poor delivery) of activity /exercise planning for the following week(s).

1 Minimal use (or poor delivery) of activity /exercise planning for the following week(s). Activities planned are not sustainable, or representative of the routine, pleasurable and necessary activities previously identified.

2 Some use of action-planning techniques using the HF Manual planning tool (or verbal equivalent) but lacking detail /patient involvement in the activity may be limited. Quality of delivery may be variable (e.g. providing the plan rather than discussing, not checking the patient is happy with the plan).

3 Appropriate use of action planning techniques . However, some difficulties evident (e.g. not summarising the plan at the end, sometimes providing rather than eliciting ideas).

4 Appropriate use of action planning techniques. Minor problems evident (e.g. the plan is a bit less specific than it could be).

5 Highly appropriate and sufficient use of action-planning techniques. The participant has a clear understanding of and ownership of the plan for the week(s) ahead. Minimal problems.

6 Excellent / expert use of action-planning techniques. The participant has a clear understanding of the rationale behind planning for the week(s) ahead, and has a clear and realistic action plan for the week(s) ahead. No real problems.

For peer review only - http://bmjopen.bmj.com/site/about/guidelines.xhtml

ITEM 7: ADDRESSING EMOTIONAL CONSEQUENCES OF HEART FAILURE

Key features: The facilitator should help the patient to recognise and address any significant stress, anxiety, anger, depression or other negative feelings that are related to having heart failure. S/he should seek to normalise such feelings and help the patient to access and work through relevant sections of the manual. If these problems are severe or prolonged the facilitator should facilitate a referral to relevant care services.

Intervention techniques: Patient centred counselling techniques (OARS) for assessment and exchanging information to build patient's understanding of the situation. Facilitation of the cognitive behavioural therapy techniques and stress management techniques contained within the manual.

Mark with an 'X' on the vertical line, using whole and half numbers, the level to which you think the facilitator has delivered this intervention process

0 Absence (or very poor delivery) of any attempts to address emotional consequences.

1 Minimal (or poorly delivered) attempts to address emotional consequences,

2 Some attempts to address emotional consequences, but lacking sufficient depth or detail. Quality of delivery may be variable (e.g. talking at odds with the patient).

3 Appropriate attempts to address emotional consequences. However, some difficulties evident (e.g. sometimes being prescriptive rather than patient-centred, not identifying all relevant sections of the manual).

4 Appropriate attempts to address emotional consequences. Minor problems evident.

5 Highly appropriate and sufficient addressing of emotional consequences. Minimal problems.

6 Excellent / expert addressing of emotional consequences. No real problems.

For peer review only - http://bmjopen.bmj.com/site/about/guidelines.xhtml

ITEM 8: ADDRESSING MEDICATION ISSUES

Key features: The facilitator should help the patient to recognise and address any significant problems or concerns relating to the patient's heart failure medications. S/he should help the patient to work through relevant sections of the manual. This might include problems in organising /taking the medications, knowing what to do if they get a cold or forget a dose, identifying possible side effects and seeking help to minimise them, avoiding over-the-counter medications. For some patients, it may include discussing self-titration of diuretics (water tablets) in response to symptoms /swelling (using the Traffic Light plan as a guide).

Intervention techniques: Patient centred counselling techniques (OARS) for assessment and to exchange information to build patient's understanding of the situation. Facilitation of medication planning /monitoring tools (in the Progress Tracker) and tips provided in the manual.

Mark with an 'X' on the vertical line, using whole and half numbers, the level to which you think the facilitator has delivered this intervention process

0 Absence (or very poor delivery) of any attempts to address medication issues.

1 Minimal (or poor delivery) attempts to address medication issues.

2 Some attempts to address medication issues, but lacking sufficient depth or detail, or quality of delivery may be variable (e.g. not picking up /addressing concerns about possible side effects)

3 Appropriate attempts to address medication issues. However, some difficulties evident (e.g. sometimes being prescriptive rather than patient-centred, not identifying all relevant sections of the manual).

4 Appropriate attempts to address medication issues. Minor problems evident.

5 Highly appropriate and sufficient addressing of medication issues. Minimal problems.

6 Excellent / expert addressing of medication issues. No real problems.

For peer review only - http://bmjopen.bmj.com/site/about/guidelines.xhtml

ITEM 9: CAREGIVER INVOLVEMENT (as applicable)

Key features: The facilitator should engage the caregiver as much as possible as a co-facilitator of the intervention. S/he should tailor the intervention to work with the caregiver's abilities and availability to provide support to the cared for person with self-management of their heart failure. Facilitators will provide the Caregiver Resource, a brief overview of what it contains, and identify some key sections for the caregiver to read.

Intervention techniques: Person centred counselling techniques (OARS) for assessment and to exchange information to build the caregiver's understanding of the situation and their ability to support the person with heart failure with their self-management. The facilitator should facilitate a conversation between the patient and the caregiver to agree their roles and responsibilities and how these might change if the patient's condition declines. Attention should be given to the caregiver's needs and concerns about being a caregiver /providing care as well as those of the patient.

Mark with an 'X' on the vertical line, using whole and half numbers, the level to which you think the facilitator has delivered this intervention process

0 Absence (or very poor delivery) of any attempts to involve the caregiver or to address his /her needs.

1 Minimal (or poor delivery) attempts to involve the caregiver or to address his /her needs.

2 Some attempts to involve the caregiver or to address his /her needs, but lacking sufficient depth or detail, or quality of delivery may be variable (e.g. being mostly prescriptive rather than person-centred).

3 Appropriate attempts to involve the caregiver or to address his /her needs. However, some difficulties evident (e.g. leaving roles and responsibilities between patient and caregiver unclear in some respects).

4 Appropriate attempts to involve the caregiver or to address his /her needs. Minor problems evident.

5 Highly appropriate and sufficient involvement of the caregiver and addressing his /her needs. Minimal problems.

6 Excellent / expert involvement of the caregiver and addressing his /her needs. No real problems.

For peer review only - http://bmjopen.bmj.com/site/about/guidelines.xhtml

ITEM 10: ADDRESSING EMOTIONAL CONSEQUENCES OF BEING A CAREGIVER (as applicable)

Key features: The facilitator should help the caregiver to recognise and address any significant stress, anxiety, anger, depression or other negative feelings that are related to becoming a caregiver and supporting someone with heart failure. S/he should seek to normalise such feelings and help the caregiver to access and work through relevant sections of the Caregiver Resource. This includes facilitating a referral for a carer's assessment if the caregiver wishes, plus referral to other relevant care services as appropriate.

Intervention techniques: Person centred counselling techniques (OARS) for assessment and to exchange information to build the caregiver's understanding of the situation. Facilitation of the cognitive behavioural therapy techniques and stress management techniques contained within the manual.

Mark with an 'X' on the vertical line, using whole and half numbers, the level to which you think the facilitator has delivered this intervention process

0 Absence (or very poor delivery) of any attempts to address emotional consequences.

1 Minimal (or poorly delivered) attempts to address emotional consequences.

2 Some attempts to address emotional consequences, but lacking sufficient depth or detail, or quality of delivery may be variable (e.g. talking at odds with the patient).

3 Appropriate attempts to address emotional consequences. However, some difficulties evident (e.g. sometimes being prescriptive rather than patient-centred, not identifying all relevant sections of the manual, not facilitating onward referrals).

4 Appropriate attempts to address emotional consequences. Minor problems evident.

5 Highly appropriate and sufficient addressing of emotional consequences. Minimal problems.

6 Excellent / expert addressing of emotional consequences. No real problems.

For peer review only - http://bmjopen.bmj.com/site/about/guidelines.xhtml

For peer review only

For peer review only - http://bmjopen.bmj.com/site/about/guidelines.xhtml

ITEM 11: CAREGIVER HEALTH AND WELL-BEING (as applicable)

Key features: The facilitator should help the caregiver to prioritise and look after their own health and well-being.

Intervention techniques: Person centred counselling techniques (OARS) for assessment and to exchange information to build the caregiver's understanding of the situation – helping them recognise and manage their own health needs including mental health, physical health, and social needs. This may be a separate conversation with the caregiver alone.

Mark with an 'X' on the vertical line, using whole and half numbers, the level to which you think the facilitator has delivered this intervention process

For peer review only

0 Absence (or very poor delivery) of any attempts to involve the caregiver or to address his /her health needs.

1 Minimal (or poor delivery of) attempts to involve the caregiver or to address his /her health needs.

2 Some attempts to involve the caregiver or to address his /her needs, but lacking sufficient depth or detail, or quality of delivery may be variable (e.g. not picking up on /addressing some of the caregiver's concerns).

3 Appropriate attempts to involve the caregiver or to address his /her needs. However, some difficulties evident (e.g. sometimes being prescriptive rather than patient-centred, failing to identify the appropriate sections of the Caregiver's Resource).

4 Appropriate attempts to involve the caregiver or to address his /her needs. Minor problems evident.

5 Highly appropriate and sufficient involvement of the caregiver and addressing his /her needs. Minimal problems.

6 Excellent / expert involvement of the caregiver and addressing his /her needs. No real problems.

For peer review only - http://bmjopen.bmj.com/site/about/guidelines.xhtml

ITEM 12: BRINGING THE PROGRAMME TO A CLOSE

Key features: Progress should be consolidated and reinforced. Plans for long-term sustainability of activities and strategies learned for managing heart failure should be discussed.

Intervention techniques: The facilitator will review progress since the start of the intervention and reinforce what has been learnt. Useful strategies that were helpful should be identified. Plans to stay well /prevent relapse should be discussed as well as 'cues for action' and plans to revisit the manual in the future. The facilitator will discuss plans to sustain any new activities, identifying any potential problems and coping strategies to overcome these. The possibility of good and bad days should be discussed and normalised.

Mark with an 'X' on the vertical line, using whole and half numbers, the level to which you think the facilitator has delivered this intervention process

0 Absence (or very poor delivery) of discussion to bring the intervention to a close. Not considering progress and long term planning using the above strategies.

1 Minimal (or poorly delivered) discussion to bring the intervention to a close. Minimal consideration of progress and long term planning using the above strategies.

2 Some discussion to bring the intervention to a close. Some consideration of progress and long term planning using the above strategies, but not in sufficient depth or detail, or quality of delivery may be variable (e.g. telling /providing solutions rather than discussing or eliciting solutions from the patient (and caregiver if relevant)).

3 Appropriate discussions to bring the intervention to a close. Appropriate consideration of progress and long term planning using the above strategies. However some difficulties evident (e.g. missed opportunities to reinforce what has been learnt, facilitator sometimes dominating the conversation /telling rather than facilitating development of the patient's own ideas).

4 Several examples of appropriate discussion to bring the intervention to a close and examples of consideration of progress and long term planning the above strategies. Minor problems evident.

For peer review only - http://bmjopen.bmj.com/site/about/guidelines.xhtml

5 Highly appropriate and sufficient discussion to bring the intervention to a close and to consider progress and long term planning using the above strategies. Minimal problems.

6 Excellent / expert discussions to bring the intervention to a close and to consider progress and long term planning using the above strategies. No real problems.

For peer review only

For peer review only - http://bmjopen.bmj.com/site/about/guidelines.xhtml

CONTENT CHECKLIST - PATIENT

**How much did the facilitator cover the following topics in this session with regard to the patient…**	**Not at all <- Partially -> Thoroughly**				
1. … Understanding heart failure	1	2	3	4	5
2. ... Management of stress or anxiety	1	2	3	4	5
3. ... Physical activity	1	2	3	4	5
4. ... Low mood /depression	1	2	3	4	5
5. … Taking medications	1	2	3	4	5
6. ... Deciding priorities/ setting goals					
7. … Tracking and reviewing progress	1	2	3	4	5
8. ... Using the HF Manual	1	2	3	4	5
9. ...Support from others	1	2	3	4	5
10. … Other (please state)	1	2	3	4	5

For peer review only

For peer review only - http://bmjopen.bmj.com/site/about/guidelines.xhtml

CONTENT CHECKLIST - CAREGIVER

How much did the facilitator cover the following topics in this session with regard to the caregiver …	Not at all <- Partially -> Thoroughly				
1. ... Assessing the caregiver's needs<br>e.g. understanding of HF, how to facilitate self care	1	2	3	4	5
2. ... Managing the caregiver's own health and well-being	1	2	3	4	5
3. ... Facilitating discussion of /decisions about care-giving roles and responsibilities	1	2	3	4	5
4. ... Promoting physical activity for the patient	1	2	3	4	5
5. ...Encouraging self-monitoring and management for the patient	1	2	3	4	5
6. ... Helping patients who feel stressed or depressed	1	2	3	4	5
7. … Understanding and managing the patient's medications	1	2	3	4	5
8. … Other (please state)<br>e.g. financial management, getting help from friends, uncertainty	1	2	3	4	5

Leventhal H, Nerenz DR, Steele DJ: Illness representations and coping with health threats. In: *Handbook of Psychology and Health*. Volume IV. Edited by Baum AE, et al. Hillsdale NJ: Lawrence Erlbaum; 1984: 219-67.

For peer review only - http://bmjopen.bmj.com/site/about/guidelines.xhtml

Dear REACH-HF facilitator,

At the end of each REACH-HF session that you have audio recorded, we would like you to take a few moments to reflect on how the session went. Each line on the checklist represents a key feature of the programme. You can rate the session from 0 to 6, where 0 means that you did not use the particular feature of the programme and 6 means that you used such feature extensively and proficiently.
There is no right or wrong way to answer these questions and your or your team's performance will not be judged in any way. We appreciate that some features will be more relevant at different points of the treatment and we do not expect you to include all features in every session. Your honesty will be greatly appreciated.

Session date: ____________________ Participant study number: ____________________ Session number: ____________________

**REACH-HF programme feature**	Absence	Minimal	Some	Sufficient	Good	Very good	Excellent
1. Active patient involvement	0	1	2	3	4	5	6
2. Assessing the patient's current situation and needs	0	1	2	3	4	5	6
3. Formulating an appropriate (individualised) treatment plan	0	1	2	3	4	5	6
4. Building the patient's understanding of heart failure /making a link between self-care activities and their heart failure symptoms	0	1	2	3	4	5	6
5a. Supporting self-monitoring and progress-tracking	0	1	2	3	4	5	6
5b. Reviewing progress and problem-solving	0	1	2	3	4	5	6
6. Making a specific action plan for physical activity, based on the activities selected by the patient	0	1	2	3	4	5	6
7. Addressing emotional consequences of heart failure	0	1	2	3	4	5	6
8. Addressing medication issues	0	1	2	3	4	5	6
9. Caregiver involvement (as applicable)	0	1	2	3	4	5	6
10. Addressing emotional consequences of being a caregiver (as applicable)	0	1	2	3	4	5	6
11. Caregiver health and well-being (as applicable)	0	1	2	3	4	5	6
12. Bringing the programme to a close	0	1	2	3	4	5	6

For peer review only

BMJ Open

Getting evidence into clinical practice: Protocol for evaluation of the implementation of a home-based cardiac rehabilitation programme for patients with heart failure.

Journal:	*BMJ Open*
Manuscript ID	bmjopen-2019-036137.R1
Article Type:	Protocol
Date Submitted by the Author:	28-Apr-2020
Complete List of Authors:	Daw, Paulina; University of Birmingham, School of Sport, Exercise & Rehabilitation Sciences<br>van Beurden, Samantha; University of Exeter, Medical School; University of Exeter, Psychology<br>Greaves, Colin; University of Birmingham, School of Sport, Exercise & Rehabilitation Sciences<br>Veldhuijzen van Zanten, Jet; University of Birmingham, School of Sport, Exercise and Rehabilitation Sciences<br>Harrison, Alexander; University of York, Health Sciences<br>Dalal, Hasnain; Royal Cornwall Hospitals NHS Trust; University of Exeter, Medical School<br>McDonagh, Sinead; University of Exeter, Medical School, Primary Care Department<br>Doherty, Patrick Joseph; University of York, Health Sciences<br>Taylor, Rod; University of Glasgow, MRC/CSO Social and Public Health Sciences Unit & Robertson Centre for Biostatistics, Institute of Health and Well Being; University of Exeter, College of Medicine and Health
**Primary Subject Heading**:	Rehabilitation medicine
Secondary Subject Heading:	Cardiovascular medicine, Evidence based practice, Health services research, Qualitative research
Keywords:	Heart failure < CARDIOLOGY, REHABILITATION MEDICINE, Change management < HEALTH SERVICES ADMINISTRATION & MANAGEMENT

SCHOLARONE™ Manuscripts

I, the Submitting Author has the right to grant and does grant on behalf of all authors of the Work (as defined in the below author licence), an exclusive licence and/or a non-exclusive licence for contributions from authors who are: i) UK Crown employees; ii) where BMJ has agreed a CC-BY licence shall apply, and/or iii) in accordance with the terms applicable for US Federal Government officers or employees acting as part of their official duties; on a worldwide, perpetual, irrevocable, royalty-free basis to BMJ Publishing Group Ltd ("BMJ") its licensees and where the relevant Journal is co-owned by BMJ to the co-owners of the Journal, to publish the Work in this journal and any other BMJ products and to exploit all rights, as set out in our licence.

The Submitting Author accepts and understands that any supply made under these terms is made by BMJ to the Submitting Author unless you are acting as an employee on behalf of your employer or a postgraduate student of an affiliated institution which is paying any applicable article publishing charge ("APC") for Open Access articles. Where the Submitting Author wishes to make the Work available on an Open Access basis (and intends to pay the relevant APC), the terms of reuse of such Open Access shall be governed by a Creative Commons licence – details of these licences and which Creative Commons licence will apply to this Work are set out in our licence referred to above.

Other than as permitted in any relevant BMJ Author's Self Archiving Policies, I confirm this Work has not been accepted for publication elsewhere, is not being considered for publication elsewhere and does not duplicate material already published. I confirm all authors consent to publication of this Work and authorise the granting of this licence.

For peer review only

Getting evidence into clinical practice: Protocol for evaluation of the implementation of a home-based cardiac rehabilitation programme for patients with heart failure.

Paulina Daw (1st author and corresponding author)
School of Sport, Exercise & Rehabilitation Sciences
University of Birmingham
Edgbaston
B15 2TT
Mobile: 0759 555 0720
Email address: pxd891@student.bham.ac.uk
ORCID iD: 0000-0002-0942-3953

Samantha Barbara van Beurden (1st author)
University of Exeter Medical School
&
Psychology
University of Exeter

PD and SVB contributed equally to this paper.

Co-authors:
Colin J Greaves
School of Sport, Exercise & Rehabilitation Sciences
University of Birmingham
Birmingham, UK

Jet JCS Veldhuijzen van Zanten
School of Sport, Exercise & Rehabilitation Sciences
University of Birmingham
Birmingham, UK

Alexander Harrison
Health Sciences
University of York, UK

Hasnain Dalal
University of Exeter Medical School
Exeter, UK
&
Royal Cornwall Hospitals NHS Trust
Cornwall, UK

Sinead TJ McDonagh
Primary Care Department
University of Exeter Medical School
Exeter, UK

Patrick J Doherty
Health Sciences
University of York
York, UK

For peer review only

Rod S Taylor
MRC/CSO Social and Public Health Sciences Unit & Robertson Centre for Biostatistics
Institute of Health and Well Being
University of Glasgow
Glasgow, UK
&
College of Medicine and Health
University of Exeter
Exeter, UK

Word count: 4324

For peer review only

ABSTRACT

Introduction: Cardiac rehabilitation (CR) improves health-related quality of life (HRQoL) and reduces hospital admissions. However, heart failure (HF) patients often fail to attend centre-based CR programmes. Novel ways of delivering healthcare, such as home-based CR programmes, may improve uptake of CR. Rehabilitation EnAblement in CHronic Heart Failure (REACH-HF) is a new, effective and cost-effective home-based CR programme for people with HF.

The aim of this prospective mixed-methods implementation evaluation study is to assess the implementation of the REACH-HF CR programme in the United Kingdom (UK) National Health Service (NHS). The specific objectives are to a) explore NHS staff perceptions of the barriers and facilitators to the implementation of REACH-HF, b) assess the quality of delivery of the programme in real-life clinical settings c) consider the nature of any adaptation(s) made and how they might impact on intervention effectiveness, and d) compare real-world patient outcomes to those seen in a prior clinical trial.

Methods and analysis: REACH-HF will be rolled-out in four NHS CR centres across the UK. Three healthcare professionals from each site will be trained to deliver the 12-week programme. In-depth qualitative interviews and focus groups will be conducted with approximately 24 NHS professionals involved in delivering or commissioning the programme. Consultations for 48 patients (12 per site) will be audio-recorded and scored using an intervention fidelity checklist. Outcomes routinely recorded in the National Audit of Cardiac Rehabilitation will be analysed and compared with outcomes from a recent randomised controlled trial: the Minnesota Living with HF Questionnaire and exercise capacity (Incremental Shuttle Walk Test). Qualitative research findings will be mapped onto the Normalisation Process Theory framework and presented in the form of a narrative synthesis. Results of the study will inform national roll-out of REACH-HF.

Ethics and dissemination:

The study (IRAS 261723) has received ethics approval from the South Central (Hampshire B) Research Ethics Committee (19/SC/0304). Written informed consent will be obtained from all health professionals and patients participating in the study.

The research team will ensure that the study is conducted in accordance with the Declaration of Helsinki, the Data Protection Act 2018, General Data Protection Regulations, and in accordance with the Research Governance Framework for Health and Social Care (2005).

Findings will be published in scientific peer-reviewed journals and presented at local, national and international meetings to publicise and explain the research methods and findings to key audiences to facilitate the further uptake of the REACH-HF intervention.

Keywords: cardiac rehabilitation, heart failure, implementation science, Normalisation Process Theory, REACH-HF.

For peer review only

ARTICLE SUMMARY

Strengths and limitations of this study:

- This will be the first study to investigate the real-world implementation of a home-based cardiac rehabilitation programme in the UK and also include the evaluation of the real-world clinical effectiveness of the programme.
- The study will use Normalisation Process Theory as a theoretical framework to guide data collection and interpretation.
- The qualitative findings will inform the development of an implementation manual for policy-makers, planners, providers and commissioners of cardiac rehabilitation services for heart failure patients.
- A possible limitation of the study is that the four centres that will be appointed to implement REACH-HF are large, well-established cardiac rehabilitation treatment centres and might not be representative of the national cardiac rehabilitation landscape – a potential sample bias towards early adopters.
- This study may have limited generalisability outside the UK.

Study registration: not applicable.

For peer review only

INTRODUCTION

Heart failure

Approximately 900,000 people are affected by heart failure (HF) in the United Kingdom (UK).[1] Due to an ageing population, HF is becoming a national healthcare challenge.[2] HF has a high impact on both patients and society; it can reduce exercise tolerance and health-related quality of life (HRQoL), increase the risk of mortality and unplanned hospital admissions and is associated with high healthcare costs.[3] There is also a considerable burden on the friends and family of people with HF.[4] Exercise-based cardiac rehabilitation (CR) programmes have been shown to enhance HRQoL in patients with HF and reduce unplanned hospital admissions.[3,5] With sufficient adherence, these benefits are consistently achieved in trial settings with both centre- and home-based CR.[3] Although the National Institute of Health and Care Excellence (NICE) recommends that all patients with HF receive CR,[6] due to the frailty and poor health of this clinical population, as well as dislike of group-based exercise and practical constrains (e.g. transportation), participation in centre-based CR remains poor.[7] Underutilisation of CR amongst this clinical population has been highlighted in the 2010 NICE guideline, with the uptake of CR being much lower than predicted and estimated at 5.3%.[8]

REACH-HF

The Rehabilitation EnAblement for CHronic Heart Failure (REACH-HF) programme is a new CR programme for HF patients and their caregivers, aimed at achieving better HRQoL in the comfort of the patient's home. The 12-week, facilitated, home-based intervention was co-developed with patients, caregivers and clinicians,[9] using Intervention Mapping approach.[10] In recent randomised controlled trials (RCTs), REACH-HF resulted in significant clinical improvements in HRQoL and was cost-effective, with a cost falling within the current National Health Service (NHS) tariff for CR in the UK.[11,12] REACH-HF therefore provides an affordable, evidence-based, patient-centred alternative to centre-based CR. This provides a way to address the latest NICE guidance recommendation that HF patients are offered "a personalised, exercise-based cardiac rehabilitation programme in a format and setting (at home, in the community or in the hospital) that is easily accessible for the person".[6]

Implementation science: negotiating the research-to-practice gap

Research and development within the NHS is world leading. However, the NHS falls short when scaling up well-evidenced innovations or good practice.[13] The spread of innovations and evidence-based interventions across the NHS and other health care systems is subject to various challenges.[14] Firstly, moving complex interventions from research settings to real-world clinical implementation is a slow process.[15] Some of the barriers slowing down this process include the characteristics of the intervention itself such as its usability or fit with the existing processes in the organisation. Beyond this, individual or organisational barriers include the attitudes towards change and the innovation itself, resources available, expertise, time and competing priorities.[16]

Secondly, following uptake, the same intervention does not always perform in exactly the same way across different organisations. For example, there may be differences in the characteristics of the people involved. In clinical trials patients tend to be included based on predetermined criteria and

For peer review only

such criteria are rigorously checked prior to study participation. However, in practice a broader patient population may end up using the intervention. There may also be differences in the characteristics of the organisations delivering the intervention in terms of access to resources, staff and expertise, compared with those available in clinical trials. With these differences in population characteristics and access to resources, unplanned adaptations may occur to better fit the new context. This initially slows down the process of implementation, but also means that the intervention is no longer delivered as it was under clinical trial conditions.[17] Such unplanned adaptations often result in the interventions initially failing to reproduce the results that are found within the context of RCTs.[18] With a varied and ever changing healthcare landscape, it is crucial to understand the full complexity of implementing innovations into real-world clinical practice.[19] It is particularly important to explore how much of the intervention can or cannot change (and in what ways) without jeopardising the benefits of the intervention.[20]

Healthcare evaluations and improvement projects often consider performance at the level of the individual healthcare professional,[21] targeting the professional's knowledge, routines and attitudes.[22] However, there is a need for wider-reaching systems-level evaluations of the implementation process that also take into account community, organisational, system and policy level influences.[23]

Overall, implementation science aims to examine the process of implementation of healthcare innovations, in particular, the barriers and facilitators, as observed in real-life clinical settings.[24] To narrow the research-to-practice gap, implementation scientists recommend that the process of implementation is considered and built into the intervention design and development, the context and systems of implementation are assessed during the implementation efforts and key stakeholders are involved in the intervention development stage through to dissemination, implementation and evaluation.[23]

Aims of the project

The current project aims to implement REACH-HF in four UK NHS CR services to a) explore the facilitators of, and barriers to, implementation of REACH-HF in existing UK CR services, b) assess the implementation fidelity and c) the extent and nature of any potential adaptations to the intervention content and how such adaptations impact on effectiveness, and d) compare real-world outcomes to the clinical trial findings.

METHODS AND ANALYSIS

Design

We will conduct a mixed-methods implementation evaluation study using in-depth semi-structured interviews with key NHS staff, analysis of pre-post intervention changes in routinely collected outcome data via the British Heart Foundation founded National Audit of Cardiac Rehabilitation (NACR) and a fidelity assessment using a checklist applied to recordings of provider-patient interactions.

In-depth semi-structured interviews will be used to identify facilitators of, and barriers to, implementation, audio-recordings of REACH-HF clinical encounters will be used to assess fidelity. Quantitative data obtained from the NACR will be used to compare real-world outcomes to the

For peer review only

clinical trial findings. Data gathered from all of the above study activities (interviews, fidelity assessment, patient outcomes) will be used to assess the extent and nature of adaptations to the intervention content and how such adaptations are associated with effectiveness.

Setting and Site Recruitment

The study will be conducted in four, UK NHS CR centres (desirably form the four UK countries) who will be early adopters of the REACH-HF programme and known as 'Beacon Sites'. The opportunity to apply to become a Beacon Site will be promoted at national (UK) conferences and local meetings of CR practitioners. Interested CR services will be sent an information pack including an application form. Applicants will be asked to provide information on their NACR National Certification Programme for CR status (NCP_CR), number of referrals made to the CR service (for both cardiac patients and patients with a primary diagnosis of HF), whether the service is offering home-based programme, length of current programmes, number of programme completions, number of pre- and post-treatment assessment completions, as well as to comment on willingness to engage in research and host site visits for other interested parties. The NCP_CR is a national certification programme for CR issued jointly by the British Association for Cardiovascular Prevention and Rehabilitation (BACPR) and the NACR. The certification programme rates CR services on seven Key Performance Indicators (KPIs). KPIs are the NACR measurable indicators based on the BACPR core components. Programmes need to meet at least four KPIs to be granted an amber status and all seven to be granted a green status (2019 NACR Quality and Outcomes report).

The sites will be recruited from across the UK using a two-stage application process (application form followed by panel interview for shortlisted sites). As an incentive, sites will be offered free intervention materials for the treatment of 50 patients (i.e., the REACH-HF patient manual, the Family & Friends Resource, audio with relaxation techniques and chair-based exercise DVD). In addition, the selected sites will be offered free training (including training manuals) for three health professionals to deliver REACH-HF, post-training support and formative feedback on performance. The three-day training will be delivered by the Heart Manual Department (HMD), NHS Lothian in Edinburgh.

To be eligible, sites have to be:

- NACR electronically registered sites with high quality status from the past audit period (green or amber status) operating in England, Wales or Northern Ireland.
- Committed to delivering REACH-HF to 50 patients over the 12-month Beacon Site project period.
- Able to release three healthcare professionals (or more) with relevant experience in CR and/or HF for three days training plus one self-directed pre-training day.
- Able to engage in research to evaluate performance (i.e., recording some intervention sessions, staff participation in interviews).
- Willing to host site visits and/or share information and/or experiences with other interested NHS parties.
- Conduct baseline and post-treatment assessment of HRQoL using the Minnesota Living with Heart Failure Questionnaire (MLHFQ)[25] and exercise capacity using the

For peer review only - http://bmjopen.bmj.com/site/about/guidelines.xhtml

Incremental Shuttle Walk Test (ISWT)[26] for all patients receiving the REACH-HF programme.

Study population

Healthcare providers: We aim to recruit up to 24 healthcare professionals. The total number will include the 12 health professionals delivering REACH-HF and other key NHS staff involved in the delivery, planning, and commissioning of CR for patients with HF. To identify key staff involved in CR services, the study will use a combination of opportunity sampling (all available staff trained to deliver the REACH-HF programme) and snowball sampling (staff who are identified by existing participants as having a key role in delivering or commissioning of CR).[27] This sampling strategy will be applied until saturation in the themes and concepts generated in the qualitative analysis is reached.

Patients: The study will include up to 200 patients with HF who are referred to the CR centres for rehabilitation and receive REACH-HF treatment. Out of the 200 patients, CR consultations of up to 48 patients (12 per site) receiving REACH-HF intervention will be audio-recorded.

Intervention

REACH-HF is a home-based, health professional facilitated, 12-week CR programme supporting self-care in patients with HF, which has been co-developed with patients, caregivers and clinicians. The programme is described in detail elsewhere,[10-12,28-31] and is summarised below.

The programme consists of:

- The Heart Failure Manual for the patient provides information about HF to increase understanding of the condition and address common misconceptions, information about and strategies for managing the condition, and further information related to HF, such as lifestyle risk management, managing depression and anxiety, and getting support from others.
- A choice of two exercise training programmes; a chair-based programme (available on DVD and online) and a walking programme. Patients are recommended to engage in exercise three times per week, in addition to general physical activity.
- A stress management programme, with relaxation techniques, provided in the manual and in audio format, to help cope with anxiety and depression.
- A Progress Tracker designed for the patient to facilitate learning from experience through self-monitoring of behaviour and symptoms – prompting help-seeking, where necessary.
- A Family and Friends Resource to increase caregiver understanding of the condition, to enable them to support the patient in their self-care, and to help them address their wellbeing.
- Face-to-face and telephone facilitation over 12 weeks by a health professional trained to deliver the REACH-HF programme.

For peer review only

Facilitator Training

Three health professionals with CR and/or HF experience from each Beacon Site will attend a three day training course delivered by the HMD in Edinburgh. This training course will focus on the 7-steps of successful facilitation of REACH-HF (Fig 1) and include sessions on psychology, behaviour change, physical activity and exercise, engaging the caregiver, and further content/interaction designed to bring all of the components together.

Figure 1. The 7-steps of successful REACH-HF facilitation.

The Beacon Sites will determine which members of the CR team will attend the REACH-HF training. The main requirement for the healthcare professional is experience of delivering CR and/or of working with HF patients. The facilitators will likely be HF/cardiac specialist nurses, or physiotherapists/exercise specialists with qualifications and/or experience in the delivery of exercise-based CR programmes.

It is expected that site identification, training and set up will take approximately six months. Following the set up period, the Beacon Sites will have 12 months to deliver REACH-HF to 50 patients, during that time qualitative interviews and audio-recordings of REACH-HF sessions for selected patients will take place. At the end of Beacon Site activity, a quantitative data download will be requested from the NACR and an interim download will be requested 9 months from the end of the study to allow piloting of data-cleaning and processing procedures (stopping short of analysis).

Measures and Procedures

Qualitative interviews

In-depth semi-structured interviews and focus groups with NHS staff. NHS staff will include REACH-HF practitioners (physiotherapists and cardiac rehabilitation nurses with experience in delivering centre-based CR, who had been trained to deliver the REACH-HF programme in a 3-day training course), service managers, clinical leads and commissioners. Interviews will take place at each Beacon Site (See Appendix 1 for the Topic Guide). Each identified staff member will, if possible, be interviewed twice (at the beginning and end of the data collection window) and one focus group will be held in each locality with identified study participants (at the midpoint of the data collection window). Interviews will be either face-to-face or by phone. The development of topic guides for qualitative interviews and focus groups was based on four constructs and 16 sub-domains from the Normalisation Process Theory (NPT) framework (Table 1). The topic guides content may be amended depending on feedback from stakeholders and the first few interviews.

NPT construct	Construct's components	Interview questions
Coherence (sense-making)	Differentiation	Can you describe REACH-HF intervention and how it differs from your usual way of working?
	Communal specification	What is your colleagues understanding of the purpose of REACH-HF intervention?
	Individual specification	How does the intervention affect the nature of your work?
	Internalisation	In your opinion, what it the value of REACH-HF intervention? To you? To your patients?

For peer review only

Cognitive participation (relational work)	Initiation	Who are the individuals (you can include yourself) that drive REACH-HF forward and get others involved? What are their roles? What are they doing to support the project?
	Enrolment	How did the team need to change in order to introduce REACH-HF?
	Legitimation	How do you feel about being involved in the REACH-HF project?
	Activation	What is the future of REACH-HF in your service? What factors can enable the integration of REACH-HF into a cardiac rehabilitation service?
Collective action (operational work)	Interactional workability	How easy or difficult has it been to integrate REACH-HF into your existing work?
	Relational integration	How has implementing REACH-HF affected working relationships within the team?
	Skills and workability	How do the skills of the staff delivering REACH-HF match the needs of the programme?
	Contextual integration	Was REACH-HF training sufficient to allow for successful implementation? If not, what other topics or skills could have been included?<br>Are there enough resources available to support the REACH-HF programme?<br>Are there any other barriers to delivering REACH-HF on your patch?
Reflexive monitoring (appraisal work)	Systematisation	Are you in any way evaluating effectiveness, usefulness or impact of REACH-HF on the service?
	Communal appraisal	Do your colleagues consider the intervention worthwhile?
	Individual appraisal	Do you consider it worthwhile?
	Reconfiguration	Can REACH-HF intervention be easily modified and improved to suit your way of working? If yes, in what way?

Table 1. Qualitative questions and their origins in the NPT construct and components.

Two video-conferencing peer supervision sessions will be available to all REACH-HF trained facilitators, provided by the HMD, as part of the REACH-HF training package. The researchers will observe, and take notes from each of these sessions.

Fidelity assessment

All REACH-HF CR treatment sessions (4-6 contacts), both face to face and phone-based, of approximately 48 consenting patients (12 per site), will be audio recorded by the healthcare professionals delivering the programme. Each REACH-HF facilitator will be requested to audio record all treatment sessions for four HF patients. The selection of which patients to include will be guided by the researchers, using a quasi-random process. Five months after the REACH-HF training, facilitators will be asked to invite all subsequent patients to take part in the study, until two willing HF patients agree to have their treatment sessions recorded. Approximately ten months after the REACH-HF training, an e-mail will be sent to repeat the invitation and audio recording process for the next two consenting patients.

The quality of delivery (intervention fidelity) of the recorded treatments will be assessed by the researcher (PD) using the same fidelity checklist used in the original REACH-HF research study.[11] This will allow comparison with fidelity scores achieved in the clinical trial. The recordings for the first six patients will also be double scored and two researchers (PD and CG) will discuss any differences in their scores to agree and 'anchor' the scoring process and minimise coder bias. If an agreement cannot be reached, a third reviewer (JVZ) will be appointed for arbitration.

The fidelity checklist is a 12-item checklist focused on identifying key delivery processes, such as the use of a patient-centred communication style, making a plan of action and encouraging self-monitoring of progress (particularly with the exercise programme). The checklist uses the Dreyfus scale of clinical skills acquisition,[32] to rate clinical skills on a scale of 0-6 and is anchored such that a score of three or more represents adequate delivery quality for each item. Coding instructions are provided (Appendix 2).

REACH-HF facilitators will be asked to complete a brief self-rated fidelity checklist after each session they have recorded. This comprises questions about the same 12 main components of the treatment and allows the facilitators to rate the occurrences of each feature (absence, minimal, some, sufficient, good, very good, excellent) (Appendix 3). The main reason for including a self-rated fidelity checklist is that an independent observer-rating is time-consuming/labour intensive, whereas a self-rating assessment might provide a pragmatic, lower-cost alternative for checking delivery quality for use in real-world clinical practice.

Lastly, for each patient opting into the study, age, sex, time since diagnosis and severity of symptoms will be recorded by the healthcare professionals delivering the REACH-HF intervention.

Quantitative

At the end of the Beacon Site project period, a report will be requested from the NACR team based at the University of York on:

- number of referrals made to the Beacon Sites during the study period,
- number of HF patients enrolled on the REACH-HF programme (attending at least one session),
- CR attendance (average number of face-to-face and telephone sessions per patient),
- number of patients completing the REACH-HF programme (in the clinical trial[11] patient adherence was defined as attendance at the first face-to-face contact with the facilitator and at least two facilitator contacts thereafter – at least one of which must have been face to face).

Summary data on key pre- and post-programme measures will also be requested to enable comparison with changes in the intervention group observed in the clinical trial. These include HRQoL – determined using the MLHFQ and exercise capacity – determined using the ISWT. The MLHFQ consist of 21 questions that rate on a scale of 0-5 (where 0 is not at all, 1 is very little and 5 is very much) how different HF symptoms (i.e., swelling of ankles and legs, shortness of breath or tiredness, fatigue and poor energy levels) prevent the patient from living as they would have wanted to during the four week period prior to the first CR session. ISWT is an externally paced exercise

For peer review only

capacity test that can be administered in the field with minimal equipment and without medical supervision. The test has good test-retest reliability and it is an acceptable alternative to (widely used to assess physical fitness and functional capacity of cardiac patients) exercise test with electrocardiogram monitoring or the cardiopulmonary exercise test.[33] A recent study confirmed that a single ISWT is a valid, low resource, assessment of an estimate for physical fitness and functional capacity for CR patients.[34]

Data Analysis

Qualitative data

Digital recordings of interviews and focus groups will be transcribed verbatim and any potentially identifiable information, such as individual or location names, will be redacted. The transcripts (Word documents) will be uploaded into NVivo software to help organise the data for analysis.[35] Illustrative quotes, that may be used in future presentations or publications, will be presented alongside pseudonyms to protect anonymity.

The transcripts will be analysed according to the principles of framework analysis outlined by Ritchie and Spencer,[36] and using the four over-arching constructs of NPT (coherence, cognitive participation, collective action and reflexive monitoring) as an initial framework for coding the data.[37] NPT suggests general mechanisms that are associated with successful implementation. These include service providers' understanding of the new intervention and how it differs from standard practice, their motivation and attitude toward the healthcare innovation and the work they do to deliver and evaluate the intervention. NPT will provide a framework for generating questions for interviews and focus groups and analysing gathered data. See Table 1 for more details on the application of NPT to the data collection.

Fidelity assessment

Implementation fidelity scores from the fidelity checklist will be collated at the level of the facilitator, the site and the total sample, and presented using descriptive statistics (means, ranges) using the same analytic approach as the original REACH-HF trial.[11] Numerical data (0-6) from the Dreyfus scale of clinical skills acquisition will be converted into categorical (yes/no) data reflecting whether the session reflected the adequate level of delivery (score three or above). Observer-rated treatment fidelity will be compared with self-rated fidelity from the post-session fidelity questionnaires completed by the REACH-HF facilitators at the end of each recorded session. The analytic approach to compare the two rating scales will be Pearson's correlation for continuous scores,[38] and Gwet's first-order agreement coefficient (the AC1 statistic) for categorical ratings.[39]

The fidelity assessment data sample reflects the sample size used to assess fidelity in the original REACH-HF clinical trial. We require a minimum of four patient recordings per facilitator to be able to assess variation in performance between staff and between NHS sites.

Quantitative outcomes

Changes from pre- to post-treatment in outcome data (MLHFQ and ISWT) will be reported as mean scores with 95 % confidence intervals within each Beacon Site. Mean change scores for patients

For peer review only

receiving REACH-HF will be compared across Beacon Sites and also with the changes found in the REACH-HF trial. This comparison will take account of potential differences on patient characteristic and take due attention to the confidence intervals. Similarly, change scores for patients receiving REACH-HF will be compared with an aggregate change score from the NACR database for those who receive other forms of CR (primarily centre-based or digital CR). Sub-group analyses will be conducted by the NACR team to determine variations in uptake and outcomes within our REACH-HF cohort by site, sex, and other characteristics of interest (e.g. area deprivation index, rurality). Data on the number of patients treated, uptake and completion rates and session attendance, will be presented using descriptive statistics. Figure 2 illustrates interactions between the study's aims and methods, and how they link with the process of ongoing evaluation and scale-up.

Figure 2. Beacon Site evaluation and embedded processes for ongoing monitoring.

Patient and public involvement

Patient preference and acceptability have been addressed extensively during the REACH-HF clinical trials.[11,12] Six patients with HF and four caregivers have been consulted and informed the design of the REACH-HF programme. Patient and public involvement in the proposed study has included involving a member of the public to read and comment on the content of the study invite letter, participant information sheet and the consent form designed for the study. Additionally, members of all CR teams involved in the study were consulted during the process of setting up the Beacon Sites on issues such as the feasibility of the study, selected outcome measures and the burden of participation in the study. At the end of the study, the final report will be shared with NHS staff at the participating Beacon Sites, allowing them to use it for service evaluation, future service planning and sharing of good practice.

DISCUSSION

The research-to-practice translation gap is well documented. It is common that evidence-based interventions are not adopted into clinical settings and do not become routine practice. To narrow the translation gap, more insight is needed into mechanisms that allow for successful implementation of effective and cost-effective interventions. To advance the field, implementation theories and mechanisms need to be tested in real-world clinical settings.

The REACH-HF Beacon Site project is a multi-faceted and interactive approach to a phased roll-out that aims to disseminate the multi-centre trial findings, increase awareness of the REACH-HF intervention and to explore replicability of the intervention in new contexts. At the time of writing this protocol, a further four Beacon sites in Scotland have been established and will also being contributing data on the implementation of REACH-HF.[40]

In line with earlier recommendations for implementation research, this study will open a channel of feedback between researchers and implementers (NHS staff), with a common goal of improved service delivery for HF patients. This study will provide an insight into the translation of the REACH-HF clinical trial findings into real-world practice and an in-depth understanding of the implementation process in the context of current NHS provision. These findings will inform the future, larger-scale implementation of REACH-HF, offer guidance to policy-makers, planners and

For peer review only

commissioners of CR services, inform adaptations to the REACH-HF training package and intervention and facilitate adoption and spread of home-based CR for HF patients in the UK.

AUTHOR CONTRIBUTIONS: All authors contributed to the idea for the study. PD and SvB drafted the manuscript. SvB lead the set up and recruitment of Beacon Sites. SMcD is overseeing the day-to-day management of the Beacon Site project. PD secured all relevant ethical approvals for the project and prepared all study documentation. CG, JVZ, HD and RT are providing project supervision and oversight. PJD and AH will coordinate access to the NACR data. AH provided statistical analysis advice. PD will acquire and analyse the data for the study. All authors provided critical revision of the manuscript for important intellectual content and approved the final draft of the protocol for submission.

FUNDING STATEMENT: This study/project was funded by the National Institute for Health Research (NIHR) [Programme Grants for Applied Research scheme (project reference RP-PG-1210-12004)]. The views expressed are those of the author(s) and not necessarily those of the NIHR or the Department of Health and Social Care.

PD's time is funded by a PhD studentship from the University of Birmingham.

COMPETING INTERESTS STATEMENT: None

REFERENCES:

1 The British Heart Foundation. Facts and Figures 2019 [available from: https://www.bhf.org.uk/what-we-do/news-from-the-bhf/contact-the-press-office/facts-and-figures, date accessed: November 2019].

2 The National Health Service (NHS). Cardiovascular disease (CVD) 2019 [available from: https://www.england.nhs.uk/ourwork/clinical-policy/cvd/, date accessed: November 2019].

3 Long L, Mordi IR, Bridges C, et al. Exercise-based cardiac rehabilitation for adults with heart failure. *Cochrane Database Syst Rev* 2019;1:cd003331.

4 Wingham J, Frost J, Britten N, et al. Needs of caregivers in heart failure management: A qualitative study. *Chronic Illn* 2015;11(4):304-19.

5 Anderson L, Taylor RS, Cardiac rehabilitation for people with heart disease: an overview of Cochrane systematic reviews. *Cochrane Database Syst Rev* 2014;12:cd011273.

6 National Institute for Health and Care Excellence. Chronic heart failure in adults: diagnosis and management (NICE Guideline 106) 2018 [available from: https://www.nice.org.uk/guidance/ng106, date accessed: November 2019].

7 National Audit of Cardiac Rehabilitation. National Audit of Cardiac Rehabilitation (NACR) Quality and Outcomes Report 2018 [available from: https://www.bhf.org.uk/informationsupport/publications/statistics/national-audit-of-cardiac-rehabilitation-quality-and-outcomes-report-2018, date accessed: November 2019].

For peer review only

8 National Institute for Health and Care Excellence (NICE). Chronic heart failure in adults: management (NICE Clinical Guideline CG108) 2010 [available from: https://www.nice.org.uk/guidance/cg108, date accessed: November 2019].

9 Greaves CJ, Wingham J, Deighan C, et al. Optimising self-care support for people with heart failure and their caregivers: development of the Rehabilitation Enablement in Chronic Heart Failure (REACH-HF) intervention using intervention mapping. *Pilot Feasibility Stud* 2016;2:37.

10 Bartholomew Eldredge LK, Markham CM, Ruiter RAC, et al. Planning Health Promotion Programs: An Intervention Mapping Approach. Hoboken: John Wiley & Sons 2016.

11 Dalal HM, Taylor RS, Jolly K, et al. The effects and costs of home-based rehabilitation for heart failure with reduced ejection fraction: The REACH-HF multicentre randomized controlled trial. *Eur J Prev Cardio* 2018;26(3):262-72.

12 Lang CC, Smith K, Wingham J, et al. A randomised controlled trial of a facilitated home-based rehabilitation intervention in patients with heart failure with preserved ejection fraction and their caregivers: the REACH-HFpEF Pilot Study. *BMJ Open* 2018;8(4):e019649.

13 Castle-Clarke S, Edwards N, Buckingham H. Falling short: Why the NHS is still struggling to make the most of new innovations. Nuffieldtrust Briefing 2017 [available from: https://www.nuffieldtrust.org.uk/research/falling-short-why-the-nhs-is-still-struggling-to-make-the-most-of-new-innovations, date accessed: November 2019].

14 Cooksey DA. Review of UK health research funding. London: HM Treasury 2006 [available from: https://assets.publishing.service.gov.uk/government/uploads/system/uploads/attachment_data/file/228984/0118404881.pdf, date accessed: November 2019].

15 Horton T, Illingworth J, Warburton W. The spread challenge: How to support the successful uptake of innovations and improvements in health care. London: Health Foundation 2018 [available from: https://www.health.org.uk/publications/the-spread-challenge, date accessed: November 2019].

16 De Silva D. What's getting in the way? Barriers to Improvement in the NHS. The Health Foundation: evidence scan 2015 [available from: https://www.health.org.uk/publications/what%E2%80%99s-getting-in-the-way-barriers-to-improvement-in-the-nhs, date accessed: November 2019].

17 Bell A, Corfield M, Davies J, et al. Collaborative transdisciplinary intervention in early years - putting theory into practice. *Child Care Health Dev* 2010;36(1):142–8.

18 Fixsen DL, Naoom SF, Blase KA, et al. Implementation Research: A Synthesis of the Literature. Tampa, FL: University of South Florida, Louis de la Parte Florida Mental Health Institute 2005.

19 Escoffery C, Lebow-Skelley E, Haardoerfer R, et al. A systematic review of adaptations of evidence-based public health interventions globally. *Implement Sci* 2018;13(1):125.

20 Breitenstein SM, Gross D, Garvey CA, et al. Implementation fidelity in community-based interventions. *Res Nurs Health* 2010;33(2):164-73.

For peer review only

21 Grol RPTM, Bosch MC, Hulscher MEJL, et al. Planning and Studying Improvement in Patient Care: The Use of Theoretical Perspectives. *Milbank Q* 2007;85(1):93-138.

22 Grimshaw JM, Thomas RE, MacLennan G, et al. Effectiveness and efficiency of guideline dissemination and implementation strategies. *Health Technol Assess* 2004;8(6):iii-iv,1-72.

23 Greenhalgh T, Robert G, Macfarlane F, et al. Diffusion of Innovations in Service Organizations: Systematic Review and Recommendations. *Milbank Q* 2004;82(4):581-629.

24 Schliep ME, Alonzo CN, Morris MA. Beyond RCTs: Innovations in research design and methods to advance implementation science. *Evid Based Commun Assess Interv* 2018;11(3-4):82-98.

25 Bilbao A, Escobar A, García-Perez L, et al. The Minnesota living with heart failure questionnaire: comparison of different factor structures. *Health Qual Life Outcomes* 2016;14:23.

26 Singh SJ, Morgan MDL, Scott S, et al. Development of a shuttle walking test of disability in patients with chronic airways obstruction. *Thorax* 1992;47(12):1019-24.

27 Marshall MN. Sampling for qualitative research. *Fam Pract* 1996;13(6):522-526.

28 Taylor RS, Hayward C, Eyre V, et al. Clinical effectiveness and cost-effectiveness of the Rehabilitation Enablement in Chronic Heart Failure (REACH-HF) facilitated self-care rehabilitation intervention in heart failure patients and caregivers: rationale and protocol for a multicentre randomised controlled trial. *BMJ Open* 2015;5(12):e009994.

29 Taylor RS, Sadler S, Dalal HM, et al. The cost effectiveness of REACH-HF and home-based cardiac rehabilitation compared with the usual medical care for heart failure with reduced ejection fraction: A decision model-based analysis. *Eur J Prev Cardiol* 2019;26(12):1252-1261.

30 Lang CC, Smith K, Wingham J, et al. A randomised controlled trial of a facilitated home-based rehabilitation intervention in patients with heart failure with preserved ejection fraction and their caregivers: the REACH-HFpEF Pilot Study. *BMJ Open* 2018;8(4):e019649.

31 Frost J, Wingham J, Britten N, et al. Home-based rehabilitation for heart failure with reduced ejection fraction: mixed methods process evaluation of the REACH-HF multicentre randomised controlled trial. *BMJ Open* 2019;9(8):e026039.

32 Dreyfus HL. The Dreyfus model of skill acquisition. In: J. Burke, eds. Competency based education and training. London: Falmer Press 1989.

33 Hanson LC, Taylor NF, McBurney H. The 10m incremental shuttle walk test is a highly reliable field exercise test for patients referred to cardiac rehabilitation: a retest reliability study. *Physiotherapy* 2016;102(3):243-8.

34 Hanson LC, McBurney H, Taylor NF. Is the 10 m incremental shuttle walk test a useful test of exercise capacity for patients referred to cardiac rehabilitation? *Eur J Cardiovasc Nurs* 2018;17(2):159-69.

35 Richards L. Handling qualitative data: a practical guide. Los Angeles: SAGE 2014.

For peer review only

36 Ritchie J, Spencer L. Qualitative data analysis for applied policy research by Jane Ritchie and Liz Spencer. In: Bryman A, Burgess RG, eds. Analysing qualitative data. London: Routledge 1994:173-194.

37 May CR, Cummings A, Girling M, et al. Using Normalization Process Theory in feasibility studies and process evaluations of complex healthcare interventions: a systematic review. *Implement Sci* 2018;13(1):80.

38 Mukaka MM. A guide to appropriate use of correlation coefficient in medical research. *Malawi Med J* 2012;24(3):69-71.

39 Gwet KL. Handbook of Inter-Rater Reliability: The Definitive Guide to Measuring the Extent of Agreement Among Raters. Gaithersburg: Advanced Analytics 2010.

40 Implementation of an evidence-based home cardiac rehabilitation programme for heart failure patients and their caregivers in Scotland (SCOT:REACH-HF) [available from: https://www.gla.ac.uk/researchinstitutes/healthwellbeing/research/mrccsosocialandpublichealthsciencesunit/programmes/complexity/complexinterventions/scotreach/, date accessed: April 2020].

For peer review only

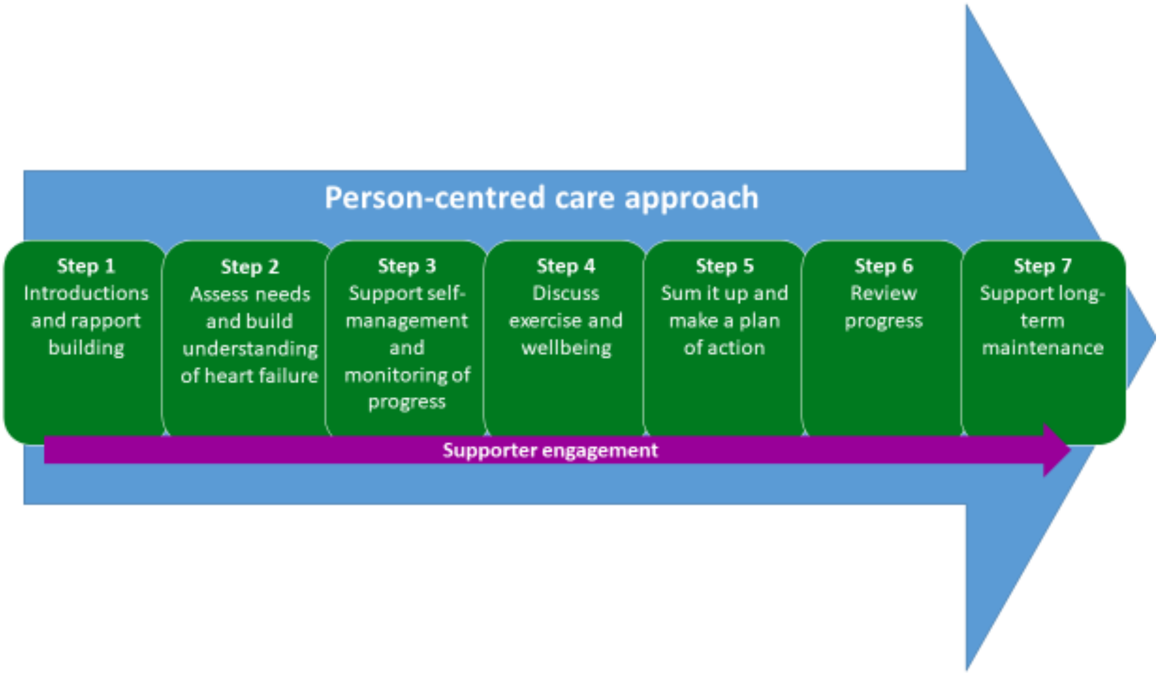

For peer review only

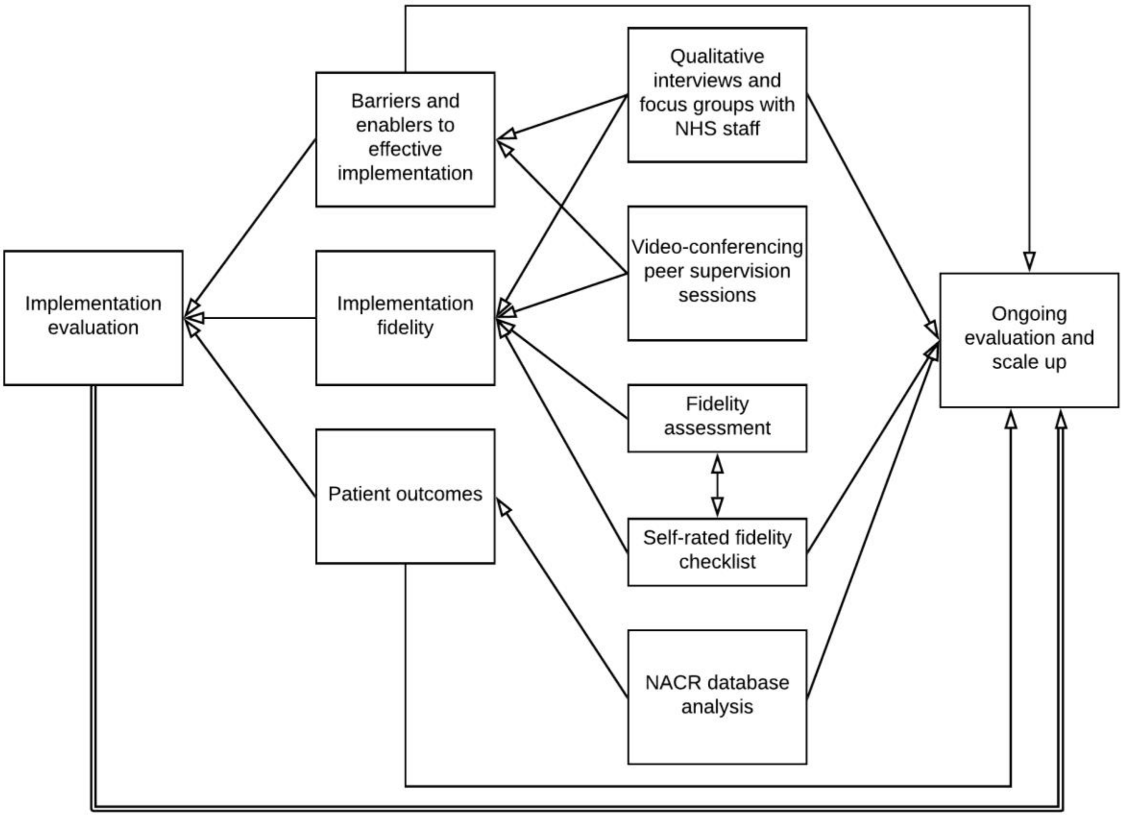
Barriers and enablers to effective implementation
Qualitative interviews and focus groups with NHS staff
Implementation evaluation
Implementation fidelity
Video-conferencing peer supervision sessions
Ongoing evaluation and scale up
Fidelity assessment
Patient outcomes
Self-rated fidelity checklist
NACR database analysis

eview only

What are the barriers and enablers to effective implementation of REACH-HF?

Qualitative interview guide (initial draft*)

** The topic guide content may vary depending on feedback from stakeholders and the first few interviews*

Beacon site: I / II / III / VI *(circle as appropriate)*

Date of interview: ____

- **Welcome and housekeeping**
  Thank you for agreeing to take part in the study. The interview will last between 30 and 40 minutes. I will ask you a series of questions and I am really interested in your honest opinion on the subject matter. If you wish to stop at any point to take a break, let me know.

- **Informed consent**
  Thank you for reading PIS and completing the consent form. Is it ok if we start recording?

- **Interview questions**

NPT	Questions	Comments
1.1	Can you describe REACH-HF intervention and how it differs from your usual way of working?	
1.3	How does the intervention affect the nature of your work?	
4.3	Do you consider it to be worthwhile?	
1.4	In your opinion what is the value of REACH-HF intervention? To you? To your patients?	
1.2	What is your colleagues understanding of the purpose of REACH-HF intervention?	
4.2	Do they consider it to be worthwhile?	
3.2	How has implementing REACH-HF affected working relationships within the team?	
2.1	Who are the individuals (you can include yourself) that drive REACH-HF forward and get others involved? What are their roles? What are they doing to support the project?	
3.1	How easy or difficult has it been to integrate REACH-HF into your existing work?	
2.2	How did the team need to change in order to introduce REACH-HF?	
2.3	How do you feel about being involved in the REACH-HF project?	
3.3	How do the skills of the staff delivering REACH-HF match the needs of the programme?	

For peer review only - http://bmjopen.bmj.com/site/about/guidelines.xhtml
For peer review only

What are the barriers and enablers to effective implementation of REACH-HF?

3.4	Was REACH-HF training sufficient to allow for successful implementation? If not, what other topics or skills could have been included?<br><br>Are there enough resources available to support the REACH-HF programme?<br><br>Are there any other **barriers** to delivering REACH-HF on your patch?	
4.1	Are you in any way evaluating effectiveness, usefulness or impact of REACH-HF on the service?	
4.4	Can REACH-HF intervention be modified and improved to suit your way of working? If yes, in what way?	
2.4	What is the future of REACH-HF in your service? What factors can **enable** integration of REACH-HF into a cardiac rehabilitation service?	

- ***A few: service-level questions:*** *What is the catchment area for your service? What population do you serve?*

- **Ending & debrief**
  Thank you for taking the time to answer my questions. Is there anything else you would like to add? Or ask me about? I am going to switch off the audio recorder now. If any of what we spoke about affected you in any way we can have a debrief session now.

For peer review only - http://bmjopen.bmj.com/site/about/guidelines.xhtml

REACH-HF FIDELITY MEASURE

The rating scale

The seven point scale extends from (0) where the facilitator did not deliver the intervention element appropriately - either they didn't do it well or didn't do it sufficiently (low fidelity) to (6) where there is the element is delivered appropriately (high fidelity). Thus the scale assesses a composite of both adherence to the intended intervention techniques and the skill of the facilitator in delivering the techniques. To aid with the rating of items, an outline of the key features of each item is provided at the top of each section. A generic description of the rating criteria is given in Figure 1.

Adjusting for the presence of patient difficulties

Adjustments may be needed when patient difficulties are evident (e.g. excessive avoidance or resistance). In such circumstances, the rater needs to assess the facilitator's therapeutic skills in the application of the methods. Even though the facilitator may not facilitate change, credit should be given for attempting to use the intended techniques and demonstrating appropriate /skilful interaction (i.e. they should do what they can, within reason, to deliver the intended intervention components).

Figure 1: The scoring system

Competence level* | **Scoring** | **Examples**

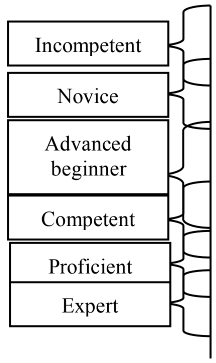

Scoring	Examples
0	Absence of feature and /or highly inappropriate performance
1	Minimal use of feature and /or inappropriate performance,
2	Evidence of competence, but numerous problems
3	Competent, but some problems or inconsistencies
4	Good features, but minor problems or inconsistencies
5	Very good features, minimal problems or inconsistencies
6	Excellent performance

* The scale incorporates the Dreyfus system (Dreyfus, 1989) for denoting competence. Please note that the 'top marks' (i.e. near the 'expert' end of the continuum) are reserved for those facilitators demonstrating highly effective skills, particularly in the face of difficulties (i.e. patients with high resistance to change; high levels of emotional expression; and complex situational barriers). Please note that there are 5 competence levels but six potential scores.

When rating the item, you should first identify whether some of the 'Key Features' are present. If the facilitator includes most of the key features and uses them appropriately (i.e. misses few relevant opportunities to use them and delivers them well), the facilitator should be rated highly. It is important to remember that the scoring profile for this scale should approximate to a normal distribution, with relatively few people

For peer review only - http://bmjopen.bmj.com/site/about/guidelines.xhtml

scoring at the extremes. **For the purposes of the REACH study, a score of 3 or more will be taken to represent "acceptable delivery or basic competence" in using the intended techniques"**

Dreyfus, H. L. (1989). The Dreyfus model of skill acquisition. In J. Burke (ed.) Competency based education and training. London: Falmer Press.

For peer review only

For peer review only - http://bmjopen.bmj.com/site/about/guidelines.xhtml

ITEM 1: ACTIVE PATIENT INVOLVEMENT

Key features: The facilitator should encourage the participant to be actively involved in the consultation. The idea is to maximise the participant's autonomy as the main agent of change, developing intrinsic rather than extrinsic motivation, and encouraging her /him to be the person coming up with ideas for improving the situation. However, the participant should not be allowed to ramble in an unstructured way and the consultation should be guided. A collaborative /shared decision-making style is appropriate and the facilitator may share his /her own expertise and ideas (as below). Overall, the participant should be increasingly empowered to take control of her /his self care behaviour. Interactions should be encouraging, respectful and non-judgemental (the opposite of a didactic, telling or persuading style of interaction). The participant should ideally talk for at least half of the time (particularly in later sessions). The interaction should also be *individually tailored* to the patient's specific information needs, beliefs, motivations and barriers. The facilitator should engender a clear sense of warmth, genuineness and empathy (within professional boundaries).

Intervention techniques: OARS (Open questions, Affirmation, Reflective listening, Summaries). Reflective listening may include simple reflections of content but may also be more sophisticated (e.g. amplified reflection; reflection with a twist) and used to direct the conversation or highlight key strengths or barriers. Summaries to reinforce patient choices and acknowledge patient effort are particularly desirable. Individual tailoring of techniques and responses to the individual patient's existing knowledge, skills, current activity levels, needs and preferences are also desirable. The Ask-Tell-Discuss technique should be used to exchange information (e.g. to address misconceptions, or offer helpful new information). The above empathy-building techniques and individual tailoring should be used throughout the consultations - from the initial consultation through action-planning through to review /maintenance sessions.

Mark with an 'X' on the vertical line, using whole and half numbers, the level to which you think the facilitator has delivered this intervention process

0 Absence of active patient involvement techniques. An overly 'directing', practitioner-led or 'lecturing' style of interaction, which may increase or sustain client's resistance.

1 Minimal patient involvement or use of active patient involvement techniques. The practitioner dominates the discussion.

2 Some use of patient involvement techniques, but not frequent enough. The practitioner sometimes dominates the discussion.

3 Appropriate and frequent use of patient involvement techniques. Teamwork evident, but some difficulties in content or method of delivery.

4 Appropriate and frequent use of patient involvement techniques. Minor problems evident (e.g. some reflection opportunities missed).

For peer review only - http://bmjopen.bmj.com/site/about/guidelines.xhtml

5 Highly appropriate and regular use of patient involvement techniques, facilitating shared understanding and decision making. Minimal problems.

6 Excellent / expert use of patient involvement techniques throughout all the consultation. A clear sense of collaborative alliance is developed.

For peer review only

For peer review only - http://bmjopen.bmj.com/site/about/guidelines.xhtml

ITEM 2: ASSESSING THE PATIENT'S CURRENT SITUATION AND NEEDS.

Key features: The facilitator should work with the participant to assess the patient's current situation. They should seek to identify ALL of the following over the first 1-2 sessions: Identify and discuss the most important issue currently for the patient, how well are they managing their fluids, how appropriately are they using medications, is there any obvious immediate clinical need, how much stress or anxiety do they have, how much physical activity are they doing, and what other concerns or questions they may have.

Intervention techniques: Facilitators will use patient-centred communication techniques (as above) which may include the Ask-Tell-Discuss and 'tell me three things' technique to explore the patient's current situation.

For peer review only

Mark with an 'X' on the vertical line, using whole and half numbers, the level to which you think the facilitator has delivered this intervention process

0 Absence (or very poor delivery) of discussions to assess the patient's current situation.

1 Minimal (or poorly delivered) discussions to assess the patient's current situation.

2 Some discussions to assess the patient's current situation, but may not be in sufficient depth or detail, or quality of delivery may be variable.

3 Several examples of discussion to assess the patient's current situation. However some difficulties evident (e.g. missed opportunities, not covering all the key topics, or talking at odds with the patient).

4 Several examples of discussion to assess the patient's current situation. Minor problems evident.

5 Highly appropriate and sufficient discussion to assess the patient's current situation. Minimal problems.

6 Excellent / expert use of discussion to assess the patient's current situation. No real problems.

For peer review only - http://bmjopen.bmj.com/site/about/guidelines.xhtml

ITEM 3: FORMULATING AN APPROPRIATE (INDIVIDUALISED) TREATMENT PLAN

Key features: The facilitator should work with the participant to formulate an appropriate treatment plan based on the patient's current situation. This should aim to address (as a minimum) ALL of the following over the twelve weeks of the programme: What is the most important issue currently for the patient, are they managing their fluids well, are they using medications appropriately, any clinical needs identified, how much stress or anxiety do they have, how much physical activity are they doing, and any other concerns or questions they may have. The treatment plan will be staged over time, aiming to work on a few topics initially and introducing other elements as the programme continues. It is best practice to summarise the treatment plan at the end of the session "what we have said today is …".

Intervention techniques: Facilitators will use patient-centred communication techniques (as above) to discuss and agree what issues to address first and what order to do things in. An element of guiding to ensure the inclusion of clinical priorities (e.g. medication issues, physical activity, psychological well-being) as well as patient priorities may be appropriate. The facilitator will advise the patient (and caregiver if appropriate) to read relevant sections of the manual ahead of their next meeting.

Mark with an 'X' on the vertical line, using whole and half numbers, the level to which you think the facilitator has delivered this intervention process

0 Absence (or very poor delivery) of discussion to formulate an appropriate treatment plan based on the patient's current situation.

1 Minimal (or poorly delivered) discussion to formulate an appropriate treatment plan based on the patient's current situation.

2 Some discussion to formulate an appropriate treatment plan based on the patient's current situation, but may not be in sufficient depth or detail, or quality of delivery may be variable (e.g. not covering all the key topics, or talking at odds with the patient).

3 Several examples of discussion to formulate an appropriate treatment plan based on the patient's current situation. However some difficulties may still be evident (e.g. missed opportunities, plan not summarised at the end of the visit).

4 Several examples of discussion to formulate an appropriate treatment plan based on the patient's current situation. Minor problems evident.

5 Highly appropriate and sufficient discussion to formulate an appropriate treatment plan based on the patient's current situation. Minimal problems.

6 Excellent / expert use of discussion to formulate an appropriate treatment plan based on the patient's current situation. No real problems.

For peer review only - http://bmjopen.bmj.com/site/about/guidelines.xhtml

ITEM 4: BUILD THE PATIENT'S UNDERSTANDING OF HEART FAILURE /MAKING A LINK BETWEEN SELF-CARE ACTIVITIES AND THEIR HEART FAILURE SYMPTOMS

Key features: Participants' ability to make sense of how HF works and how self-care behaviours might influence the course of the illness will be crucial for the success of the intervention as belief in the benefit of the suggested self-care activities will increase motivation to engage in them. The facilitator should elicit the patient's current understanding of heart failure and seek to build their 'illness model' in terms of understanding the Identity, Causes, Consequences, Cure /control options and Timeline[1] associated with the condition. This process may take several weeks and should be reinforced as the programme progresses.

Intervention techniques: Facilitators will provide the REACH-HF Manual, provide a brief overview of how the manual works and, after assessing the patient's individual needs and concerns (as above), they will identify some key sections for the patient to read before the next contact, specifically including the Understanding HF section. Facilitators will use patient-centred communication techniques (as above) to elicit and build understanding. This should include the use of the Ask-Tell-Discuss technique and reflective listening to reinforce elements of the patient's understanding that are factually correct or which predispose towards positive self-care behaviours. They should seek to reframe negative attitudes and exchange information (Ask-Tell-Discuss) to address any misconceptions or to fill any important gaps in understanding. The facilitator will advise the patient (and caregiver if appropriate) to read relevant sections of the manual (including the Understanding HF chapter) to build and reinforce understanding /to address misconceptions. The way HF works should be explicitly discussed and referred back to /reinforced at subsequent sessions when this reinforces perceived benefits of the proposed self-care behaviours.

Mark with an 'X' on the vertical line, using whole and half numbers, the level to which you think the facilitator has delivered this intervention process.

0 Absence (or very poor delivery) of any exploration or discussion of how HF works. Understanding of HF is assumed or not mentioned or discussed.

1 Minimal (or poor delivery of) exploration or discussion of how HF works.

2 Some exploration or discussion of the how HF works, but may not be in sufficient depth or detail, or quality of delivery may be variable (e.g. telling rather than Ask-Tell-Discuss) or understanding is not checked.

3 Appropriate exploration and discussion of how HF works. However, some difficulties may still be evident (e.g. moving on before understanding is fully established).

4 Appropriate exploration or discussion of how HF works, linking changes in symptoms or mood with changes in self-care behaviour. Minor problems evident (e.g. some inconsistencies).

For peer review only - http://bmjopen.bmj.com/site/about/guidelines.xhtml

5 Highly appropriate and sufficient exploration or discussion of how HF works, facilitating a clear understanding of the process and linking changes in symptoms and mood with changes in self-care behaviour. Minimal problems.

6 Excellent / expert exploration and discussion facilitating a clear understanding of how HF works and the reasons for change. No real problems.

1. Leventhal H, Nerenz DR, Steele DJ: Illness representations and coping with health threats. In: *Handbook of Psychology and Health*. Volume IV. Edited by Baum AE, et al. Hillsdale NJ: Lawrence Erlbaum; 1984: 219-67.

For peer review only

For peer review only - http://bmjopen.bmj.com/site/about/guidelines.xhtml

ITEM 5a: SUPPORTING SELF-MONITORING AND PROGRESS-TRACKING

Key features: The facilitator should agree a verbal plan of action for the following week(s) with the patient. and discuss the use of the progress-tracking tools in the HF Manual to keep track of progress and as a way of recording any problems in completing the activities and any benefits that might be associated with the planned activities.

Intervention techniques: The facilitator should encourage the participant to monitor /keep track of their activities using the progress-tracking tools in the HF Manual.

Mark with an 'X' on the vertical line, using whole and half numbers, the level to which you think the facilitator has delivered this intervention process

0 Absence (or very poor delivery) of encouragement of self-monitoring.

1 Minimal (or poorly delivered) encouragement of self-monitoring. Activities planned are not sustainable, or poorly specified.

2 Some encouragement of self-monitoring but lacking detail /patient involvement in the activity may be limited, or quality of delivery may be variable (e.g. telling rather than discussing).

3 Appropriate encouragement of self-monitoring. However, some difficulties evident (e.g. not explaining the rationale for using the tool as a basis for monitoring progress, sometimes providing rather than eliciting ideas).

4 Appropriate encouragement of self-monitoring. Minor problems evident (e.g. the plan is a bit less specific than it could be).

5 Highly appropriate encouragement of self-monitoring. The participant has a clear understanding of the plan for the week ahead and how to monitor progress. Minimal problems

6 Excellent / expert encouragement of self-monitoring. The participant has a clear and realistic understanding of how to monitor progress. No real problems.

For peer review only - http://bmjopen.bmj.com/site/about/guidelines.xhtml

ITEM 5b: REVIEWING PROGRESS AND PROBLEM-SOLVING

Key features: The facilitator should work with the participant to review progress with all planned changes and with achieving the targets set out in the action plan. The facilitator should celebrate and reinforce and reflect on any successes. The participant and facilitator should discuss any setbacks and the patient's plans should be revised.

Intervention techniques: The facilitator should reinforce any self-monitoring activity and any successes in behaviour change (by giving praise/ using Affirmation techniques). Reframing should be used to normalise setbacks and see them as an opportunity to learn from experience (trial and error) rather than as failures. Problem-solving should use OARS (Open questions, Affirmation, Reflective listening, Summaries) and information exchange (Ask-Tell-Discuss) techniques to identify barriers and explore ways to overcome them. Problem-solving may specifically focus on issues of connectedness (social influences, involvement of others in supporting activities) and sustainability, or on breaking the problem down into more manageable chunks. Goals /action plans should be reviewed and revised if necessary.

Mark with an 'X' on the vertical line, using whole and half numbers, the level to which you think the facilitator has delivered this intervention process

0 Absence (or very poor delivery) of any progress review. No reinforcement of success and discussion of setbacks or barriers in relation to the previous weeks planned activities /problem-solving, or reviewing action plans.

1 Minimal (or poor delivery) of progress review. Minimal reinforcement of success and discussion of setbacks or barriers in relation to the previous weeks planned activities /problem-solving, or reviewing action plans.

2 Some progress review. Some reinforcement of success and discussion of setbacks or barriers in relation to the previous weeks planned activities /problem-solving and reviewing action plans, but lacking sufficient depth or detail or may be poorly delivered (e.g. providing solutions rather than using Ask-Tell-Discuss).

3 Appropriate progress review. Appropriate reinforcement of success and discussion of setbacks or barriers in relation to the previous weeks planned activities /problem-solving, and reviewing action plans. However, some difficulties evident (e.g. not reframing setbacks, not attempting to identify problems, or possible solutions).

4 Appropriate progress review. Appropriate reinforcement of success and discussion of setbacks or barriers in relation to the previous weeks planned activities /problem-solving, and reviewing action plans. Minor problems evident.

5 Highly appropriate and sufficient progress review. Appropriate reinforcement of success and discussion of setbacks or barriers in relation to the previous weeks planned activities /problem-solving, or reviewing action plans. Minimal problems.

For peer review only - http://bmjopen.bmj.com/site/about/guidelines.xhtml

6 Excellent / expert progress review. Excellent reinforcement of success and discussion of setbacks or barriers in relation to the previous weeks planned activities /problem-solving, and reviewing action plans. No real problems.

For peer review only

For peer review only - http://bmjopen.bmj.com/site/about/guidelines.xhtml

ITEM 6: MAKE A SPECIFIC ACTION PLAN FOR PHYSICAL ACTIVITY, BASED ON THE ACTIVITIES SELECTED BY THE PATIENT

Key features: Using the template in the HF manual, the facilitator should work with the participant to agree a written or verbal plan of action for engaging in one of the physical activity /exercise options over the following week(s). This should include discussion to ensure an appropriate intensity (moderate) of any activity included in the action plan.

Intervention techniques: Making a written action plan, using the planning tool in the manual, or a verbal action plan for physical activity. The facilitator should ensure that goal-setting is realistic. The facilitator may also employ some problem-solving techniques at this stage to pre-empt and address potential problems. It is best practice to summarise the plan at the end of the session "what we have said today is …".

Mark with an 'X' on the vertical line, using whole and half numbers, the level to which you think the facilitator has delivered this intervention process

0 Absence (or very poor delivery) of activity /exercise planning for the following week(s).

1 Minimal use (or poor delivery) of activity /exercise planning for the following week(s). Activities planned are not sustainable, or representative of the routine, pleasurable and necessary activities previously identified.

2 Some use of action-planning techniques using the HF Manual planning tool (or verbal equivalent) but lacking detail /patient involvement in the activity may be limited. Quality of delivery may be variable (e.g. providing the plan rather than discussing, not checking the patient is happy with the plan).

3 Appropriate use of action planning techniques . However, some difficulties evident (e.g. not summarising the plan at the end, sometimes providing rather than eliciting ideas).

4 Appropriate use of action planning techniques. Minor problems evident (e.g. the plan is a bit less specific than it could be).

5 Highly appropriate and sufficient use of action-planning techniques. The participant has a clear understanding of and ownership of the plan for the week(s) ahead. Minimal problems.

6 Excellent / expert use of action-planning techniques. The participant has a clear understanding of the rationale behind planning for the week(s) ahead, and has a clear and realistic action plan for the week(s) ahead. No real problems.

For peer review only - http://bmjopen.bmj.com/site/about/guidelines.xhtml

ITEM 7: ADDRESSING EMOTIONAL CONSEQUENCES OF HEART FAILURE

Key features: The facilitator should help the patient to recognise and address any significant stress, anxiety, anger, depression or other negative feelings that are related to having heart failure. S/he should seek to normalise such feelings and help the patient to access and work through relevant sections of the manual. If these problems are severe or prolonged the facilitator should facilitate a referral to relevant care services.

Intervention techniques: Patient centred counselling techniques (OARS) for assessment and exchanging information to build patient's understanding of the situation. Facilitation of the cognitive behavioural therapy techniques and stress management techniques contained within the manual.

Mark with an 'X' on the vertical line, using whole and half numbers, the level to which you think the facilitator has delivered this intervention process

0 Absence (or very poor delivery) of any attempts to address emotional consequences.

1 Minimal (or poorly delivered) attempts to address emotional consequences,

2 Some attempts to address emotional consequences, but lacking sufficient depth or detail. Quality of delivery may be variable (e.g. talking at odds with the patient).

3 Appropriate attempts to address emotional consequences. However, some difficulties evident (e.g. sometimes being prescriptive rather than patient-centred, not identifying all relevant sections of the manual).

4 Appropriate attempts to address emotional consequences. Minor problems evident.

5 Highly appropriate and sufficient addressing of emotional consequences. Minimal problems.

6 Excellent / expert addressing of emotional consequences. No real problems.

For peer review only - http://bmjopen.bmj.com/site/about/guidelines.xhtml

ITEM 8: ADDRESSING MEDICATION ISSUES

Key features: The facilitator should help the patient to recognise and address any significant problems or concerns relating to the patient's heart failure medications. S/he should help the patient to work through relevant sections of the manual. This might include problems in organising /taking the medications, knowing what to do if they get a cold or forget a dose, identifying possible side effects and seeking help to minimise them, avoiding over-the-counter medications. For some patients, it may include discussing self-titration of diuretics (water tablets) in response to symptoms /swelling (using the Traffic Light plan as a guide).

Intervention techniques: Patient centred counselling techniques (OARS) for assessment and to exchange information to build patient's understanding of the situation. Facilitation of medication planning /monitoring tools (in the Progress Tracker) and tips provided in the manual.

For peer review only

Mark with an 'X' on the vertical line, using whole and half numbers, the level to which you think the facilitator has delivered this intervention process

0 Absence (or very poor delivery) of any attempts to address medication issues.

1 Minimal (or poor delivery) attempts to address medication issues.

2 Some attempts to address medication issues, but lacking sufficient depth or detail, or quality of delivery may be variable (e.g. not picking up /addressing concerns about possible side effects)

3 Appropriate attempts to address medication issues. However, some difficulties evident (e.g. sometimes being prescriptive rather than patient-centred, not identifying all relevant sections of the manual).

4 Appropriate attempts to address medication issues. Minor problems evident.

5 Highly appropriate and sufficient addressing of medication issues. Minimal problems.

6 Excellent / expert addressing of medication issues. No real problems.

For peer review only - http://bmjopen.bmj.com/site/about/guidelines.xhtml

ITEM 9: CAREGIVER INVOLVEMENT (as applicable)

Key features: The facilitator should engage the caregiver as much as possible as a co-facilitator of the intervention. S/he should tailor the intervention to work with the caregiver's abilities and availability to provide support to the cared for person with self-management of their heart failure. Facilitators will provide the Caregiver Resource, a brief overview of what it contains, and identify some key sections for the caregiver to read.

Intervention techniques: Person centred counselling techniques (OARS) for assessment and to exchange information to build the caregiver's understanding of the situation and their ability to support the person with heart failure with their self-management. The facilitator should facilitate a conversation between the patient and the caregiver to agree their roles and responsibilities and how these might change if the patient's condition declines. Attention should be given to the caregiver's needs and concerns about being a caregiver /providing care as well as those of the patient.

Mark with an 'X' on the vertical line, using whole and half numbers, the level to which you think the facilitator has delivered this intervention process

0 Absence (or very poor delivery) of any attempts to involve the caregiver or to address his /her needs.

1 Minimal (or poor delivery) attempts to involve the caregiver or to address his /her needs.

2 Some attempts to involve the caregiver or to address his /her needs, but lacking sufficient depth or detail, or quality of delivery may be variable (e.g. being mostly prescriptive rather than person-centred).

3 Appropriate attempts to involve the caregiver or to address his /her needs. However, some difficulties evident (e.g. leaving roles and responsibilities between patient and caregiver unclear in some respects).

4 Appropriate attempts to involve the caregiver or to address his /her needs. Minor problems evident.

5 Highly appropriate and sufficient involvement of the caregiver and addressing his /her needs. Minimal problems.

6 Excellent / expert involvement of the caregiver and addressing his /her needs. No real problems.

For peer review only - http://bmjopen.bmj.com/site/about/guidelines.xhtml

ITEM 10: ADDRESSING EMOTIONAL CONSEQUENCES OF BEING A CAREGIVER (as applicable)

Key features: The facilitator should help the caregiver to recognise and address any significant stress, anxiety, anger, depression or other negative feelings that are related to becoming a caregiver and supporting someone with heart failure. S/he should seek to normalise such feelings and help the caregiver to access and work through relevant sections of the Caregiver Resource. This includes facilitating a referral for a carer's assessment if the caregiver wishes, plus referral to other relevant care services as appropriate.

Intervention techniques: Person centred counselling techniques (OARS) for assessment and to exchange information to build the caregiver's understanding of the situation. Facilitation of the cognitive behavioural therapy techniques and stress management techniques contained within the manual.

Mark with an 'X' on the vertical line, using whole and half numbers, the level to which you think the facilitator has delivered this intervention process

0 Absence (or very poor delivery) of any attempts to address emotional consequences.

1 Minimal (or poorly delivered) attempts to address emotional consequences.

2 Some attempts to address emotional consequences, but lacking sufficient depth or detail, or quality of delivery may be variable (e.g. talking at odds with the patient).

3 Appropriate attempts to address emotional consequences. However, some difficulties evident (e.g. sometimes being prescriptive rather than patient-centred, not identifying all relevant sections of the manual, not facilitating onward referrals).

4 Appropriate attempts to address emotional consequences. Minor problems evident.

5 Highly appropriate and sufficient addressing of emotional consequences. Minimal problems.

6 Excellent / expert addressing of emotional consequences. No real problems.

For peer review only - http://bmjopen.bmj.com/site/about/guidelines.xhtml

For peer review only

For peer review only - http://bmjopen.bmj.com/site/about/guidelines.xhtml

ITEM 11: CAREGIVER HEALTH AND WELL-BEING (as applicable)

Key features: The facilitator should help the caregiver to prioritise and look after their own health and well-being.

Intervention techniques: Person centred counselling techniques (OARS) for assessment and to exchange information to build the caregiver's understanding of the situation – helping them recognise and manage their own health needs including mental health, physical health, and social needs. This may be a separate conversation with the caregiver alone.

Mark with an 'X' on the vertical line, using whole and half numbers, the level to which you think the facilitator has delivered this intervention process

0 Absence (or very poor delivery) of any attempts to involve the caregiver or to address his /her health needs.

1 Minimal (or poor delivery of) attempts to involve the caregiver or to address his /her health needs.

2 Some attempts to involve the caregiver or to address his /her needs, but lacking sufficient depth or detail, or quality of delivery may be variable (e.g. not picking up on /addressing some of the caregiver's concerns).

3 Appropriate attempts to involve the caregiver or to address his /her needs. However, some difficulties evident (e.g. sometimes being prescriptive rather than patient-centred, failing to identify the appropriate sections of the Caregiver's Resource).

4 Appropriate attempts to involve the caregiver or to address his /her needs. Minor problems evident.

5 Highly appropriate and sufficient involvement of the caregiver and addressing his /her needs. Minimal problems.

6 Excellent / expert involvement of the caregiver and addressing his /her needs. No real problems.

For peer review only - http://bmjopen.bmj.com/site/about/guidelines.xhtml

ITEM 12: BRINGING THE PROGRAMME TO A CLOSE

Key features: Progress should be consolidated and reinforced. Plans for long-term sustainability of activities and strategies learned for managing heart failure should be discussed.

Intervention techniques: The facilitator will review progress since the start of the intervention and reinforce what has been learnt. Useful strategies that were helpful should be identified. Plans to stay well /prevent relapse should be discussed as well as 'cues for action' and plans to revisit the manual in the future. The facilitator will discuss plans to sustain any new activities, identifying any potential problems and coping strategies to overcome these. The possibility of good and bad days should be discussed and normalised.

Mark with an 'X' on the vertical line, using whole and half numbers, the level to which you think the facilitator has delivered this intervention process

0 Absence (or very poor delivery) of discussion to bring the intervention to a close. Not considering progress and long term planning using the above strategies.

1 Minimal (or poorly delivered) discussion to bring the intervention to a close. Minimal consideration of progress and long term planning using the above strategies.

2 Some discussion to bring the intervention to a close. Some consideration of progress and long term planning using the above strategies, but not in sufficient depth or detail, or quality of delivery may be variable (e.g. telling /providing solutions rather than discussing or eliciting solutions from the patient (and caregiver if relevant)).

3 Appropriate discussions to bring the intervention to a close. Appropriate consideration of progress and long term planning using the above strategies. However some difficulties evident (e.g. missed opportunities to reinforce what has been learnt, facilitator sometimes dominating the conversation /telling rather than facilitating development of the patient's own ideas).

4 Several examples of appropriate discussion to bring the intervention to a close and examples of consideration of progress and long term planning the above strategies. Minor problems evident.

For peer review only

For peer review only - http://bmjopen.bmj.com/site/about/guidelines.xhtml

5 Highly appropriate and sufficient discussion to bring the intervention to a close and to consider progress and long term planning using the above strategies. Minimal problems.

6 Excellent / expert discussions to bring the intervention to a close and to consider progress and long term planning using the above strategies. No real problems.

For peer review only

For peer review only - http://bmjopen.bmj.com/site/about/guidelines.xhtml

CONTENT CHECKLIST - PATIENT

**How much did the facilitator cover the following topics in this session with regard to the patient…**	**Not at all Thoroughly**	**<-**	**Partially**	**->**	
1. … Understanding heart failure	1	2	3	4	5
2. ... Management of stress or anxiety	1	2	3	4	5
3. ... Physical activity	1	2	3	4	5
4. ... Low mood /depression	1	2	3	4	5
5. … Taking medications	1	2	3	4	5
6. ... Deciding priorities/ setting goals					
7. … Tracking and reviewing progress	1	2	3	4	5
8. ... Using the HF Manual	1	2	3	4	5
9. ...Support from others	1	2	3	4	5
10. … Other (please state)	1	2	3	4	5

For peer review only

For peer review only - http://bmjopen.bmj.com/site/about/guidelines.xhtml

CONTENT CHECKLIST - CAREGIVER

**How much did the facilitator cover the following topics in this session with regard to the caregiver …**	**Not at all <- Partially -> Thoroughly**				
1. ... Assessing the caregiver's needs<br>e.g. understanding of HF, how to facilitate self care	1	2	3	4	5
2. ... Managing the caregiver's own health and well-being	1	2	3	4	5
3. ... Facilitating discussion of /decisions about care-giving roles and responsibilities	1	2	3	4	5
4. ... Promoting physical activity for the patient	1	2	3	4	5
5. ...Encouraging self-monitoring and management for the patient	1	2	3	4	5
6. ... Helping patients who feel stressed or depressed	1	2	3	4	5
7. … Understanding and managing the patient's medications	1	2	3	4	5
8. … Other (please state)<br>e.g. financial management, getting help from friends, uncertainty	1	2	3	4	5

Leventhal H, Nerenz DR, Steele DJ: Illness representations and coping with health threats. In: *Handbook of Psychology and Health*. Volume IV. Edited by Baum AE, et al. Hillsdale NJ: Lawrence Erlbaum; 1984: 219-67.

For peer review only - http://bmjopen.bmj.com/site/about/guidelines.xhtml
For peer review only

Dear REACH-HF facilitator,

At the end of each REACH-HF session that you have audio recorded, we would like you to take a few moments to reflect on how the session went. Each line on the checklist represents a key feature of the programme. You can rate the session from 0 to 6, where 0 means that you did not use the particular feature of the programme and 6 means that you used such feature extensively and proficiently.
There is no right or wrong way to answer these questions and your or your team's performance will not be judged in any way. We appreciate that some features will be more relevant at different points of the treatment and we do not expect you to include all features in every session. Your honesty will be greatly appreciated.

Session date: ____________________ Participant study number: ____________________ Session number: ____________________

**REACH-HF programme feature**	Absence	Minimal	Some	Sufficient	Good	Very good	Excellent
1. Active patient involvement	0	1	2	3	4	5	6
2. Assessing the patient's current situation and needs	0	1	2	3	4	5	6
3. Formulating an appropriate (individualised) treatment plan	0	1	2	3	4	5	6
4. Building the patient's understanding of heart failure /making a link between self-care activities and their heart failure symptoms	0	1	2	3	4	5	6
5a. Supporting self-monitoring and progress-tracking	0	1	2	3	4	5	6
5b. Reviewing progress and problem-solving	0	1	2	3	4	5	6
6. Making a specific action plan for physical activity, based on the activities selected by the patient	0	1	2	3	4	5	6
7. Addressing emotional consequences of heart failure	0	1	2	3	4	5	6
8. Addressing medication issues	0	1	2	3	4	5	6
9. Caregiver involvement (as applicable)	0	1	2	3	4	5	6
10. Addressing emotional consequences of being a caregiver (as applicable)	0	1	2	3	4	5	6
11. Caregiver health and well-being (as applicable)	0	1	2	3	4	5	6
12. Bringing the programme to a close	0	1	2	3	4	5	6

For peer review only
